# The plasticity of NBS resistance genes in sorghum is driven by multiple evolutionary processes

**DOI:** 10.1186/s12870-014-0253-z

**Published:** 2014-09-26

**Authors:** Emma Mace, Shuaishuai Tai, David Innes, Ian Godwin, Wushu Hu, Bradley Campbell, Edward Gilding, Alan Cruickshank, Peter Prentis, Jun Wang, David Jordan

**Affiliations:** Department of Agriculture, Fisheries & Forestry (DAFF), Warwick, QLD Australia; BGI-Shenzhen, Shenzhen, China; DAFFQ, Cooper’s Plains, Brisbane, QLD Australia; The University of Queensland, School of Agriculture and Food Sciences, Brisbane, QLD Australia; The Institute of Molecular Biosciences, The University of Queensland, Brisbane, QLD Australia; Queensland University of Technology, Brisbane, QLD Australia; Department of Biology, University of Copenhagen, DK-2200 Copenhagen, Denmark; The Novo Nordisk Foundation Center for Basic Metabolic Research, University of Copenhagen, DK-2200 Copenhagen, Denmark; Queensland Alliance for Agriculture and Food Innovation, The University of Queensland, Warwick, QLD Australia

**Keywords:** Cereals, Disease resistance, Domestication, Maize, NBS-LRR genes, QTL, Rice, Selection, Sorghum

## Abstract

**Background:**

Increased disease resistance is a key target of cereal breeding programs, with disease outbreaks continuing to threaten global food production, particularly in Africa. Of the disease resistance gene families, the nucleotide-binding site plus leucine-rich repeat (NBS-LRR) family is the most prevalent and ancient and is also one of the largest gene families known in plants. The sequence diversity in NBS-encoding genes was explored in sorghum, a critical food staple in Africa, with comparisons to rice and maize and with comparisons to fungal pathogen resistance QTL.

**Results:**

In sorghum, NBS-encoding genes had significantly higher diversity in comparison to non NBS-encoding genes and were significantly enriched in regions of the genome under purifying and balancing selection, both through domestication and improvement. Ancestral genes, pre-dating species divergence, were more abundant in regions with signatures of selection than in regions not under selection. Sorghum NBS-encoding genes were also significantly enriched in the regions of the genome containing fungal pathogen disease resistance QTL; with the diversity of the NBS-encoding genes influenced by the type of co-locating biotic stress resistance QTL.

**Conclusions:**

NBS-encoding genes are under strong selection pressure in sorghum, through the contrasting evolutionary processes of purifying and balancing selection. Such contrasting evolutionary processes have impacted ancestral genes more than species-specific genes. Fungal disease resistance hot-spots in the genome, with resistance against multiple pathogens, provides further insight into the mechanisms that cereals use in the “arms race” with rapidly evolving pathogens in addition to providing plant breeders with selection targets for fast-tracking the development of high performing varieties with more durable pathogen resistance.

**Electronic supplementary material:**

The online version of this article (doi:10.1186/s12870-014-0253-z) contains supplementary material, which is available to authorized users.

## Background

The grasses, including the major cereals wheat, barley, maize, rice and sorghum, are the most agronomically and economically important species, collectively feeding over two thirds of the world population [1]. However the production of these crops is challenged by pathogens which pose a major threat to the global human food supply. At least 30% of global food production is lost to pathogens [2,3] and the impact of disease outbreaks can be particularly acute in developing countries [4]. Amongst the cereals, sorghum which provides staple food for over 500 million people in the semi-arid tropics of Africa and Asia, in addition to being an important source of feed for livestock, is one of the best adapted to drought and high temperatures, and will play an increasingly important role in meeting the challenges of feeding the world’s growing population. However, its productivity is often impacted by foliar fungal diseases. The most profitable and sustainable disease minimisation strategy is to grow genetically resistant varieties; consequently, selection for disease resistance is a critical component of nearly all plant breeding programs.

Among all disease resistance genes, the nucleotide-binding site plus leucine-rich repeat genes (NBS-LRR) are the most prevalent and ancient and are one of the largest gene families known in plants [5]. These genes are involved in the detection and response to diverse pathogens, including bacteria, viruses, fungi, nematodes, insects and oomycetes [5]. NBS-LRR genes encode an N-terminal variable domain, a central nucleotide-binding site (NBS) domain, and a C-terminal leucine-rich repeat (LRR) domain [6]. Further, classification based on the presence of an N-terminal Toll/interleukin-1 receptor (TIR) domain divides NBS-encoding genes into TIR and non-TIR subclass, though previous studies have shown that the TIR subclass is under-represented in the cereals, and in monocotyledonous plants in general [7,8].

The most striking structural feature of the NBS-encoding genes is the variable number of LRR domains, with some genes lacking LRR-coding domains completely [8]. These domains are highly variable regions thought to be responsible for recognising pathogen-encoded ligands [9]. In contrast, the NBS domain, involved in signalling, includes several highly conserved and strictly ordered motifs [10].

Previous studies have identified highly variable numbers of NBS-encoding genes across plant genomes, e.g. ranging from approximately 150 in arabidopsis [11] to almost 500 in rice [12], with sorghum reported as having between 211 [13] and 348 [14] NBS-LRR genes. It has been postulated that such rapid copy number evolution is driven by gene loss or expansion within a species through repeated cycles of duplication, divergence and eventual loss by pseudogene formation or deletion in response to diverse pathogens [15]. These genes are expected to be under continual selection pressure for alleles that allow the plant to defend against pathogen attack. Initial studies have shown that NBS-encoding genes are more often the target of selection than non-NBS-encoding genes [16], but these NBS-encoding genes show molecular evidence consistent with the action of different types of selection. Some evolve relatively slowly whereas others exhibit typical patterns associated with rapid evolution, including multiple and variable copy number, a high ratio of non-synonymous to synonymous substitutions and high levels of within species polymorphism [17].

The availability of the whole genome sequences of a number of cereal crops [13,18,19] has given rise to a suite of new studies assessing genome-wide sequence polymorphism within species [20-22]. A recent resequencing study in sorghum [23] generated high-coverage (>20×) data for a diverse group of 44 wild, weedy and cultivated genotypes, spanning the dimensions of geographic origin, crop management and subgroup/race. This study utilises this resource which provides new opportunities to explore the evolutionary plasticity and resulting variability in NBS-encoding genes in sorghum wild and weedy genotypes in contrast to cultivated genotypes with respect to 1) previously identified genomic regions under selection during domestication and improvement; 2) sorghum fungal pathogen disease resistance QTL and 3) ancestral gene families shared with maize and rice. Such insights will shed new light on how the NBS-encoding gene family became the foremost pathogen surveillance system in cereal genomes. It will additionally provide breeders with new knowledge and tools for estimating the richness of resistance germplasm and targeting specific genomic regions in order to utilise these resources more efficiently.

## Results

### Polymorphism patterns in NBS-encoding genes in sorghum

**Figure 1:**
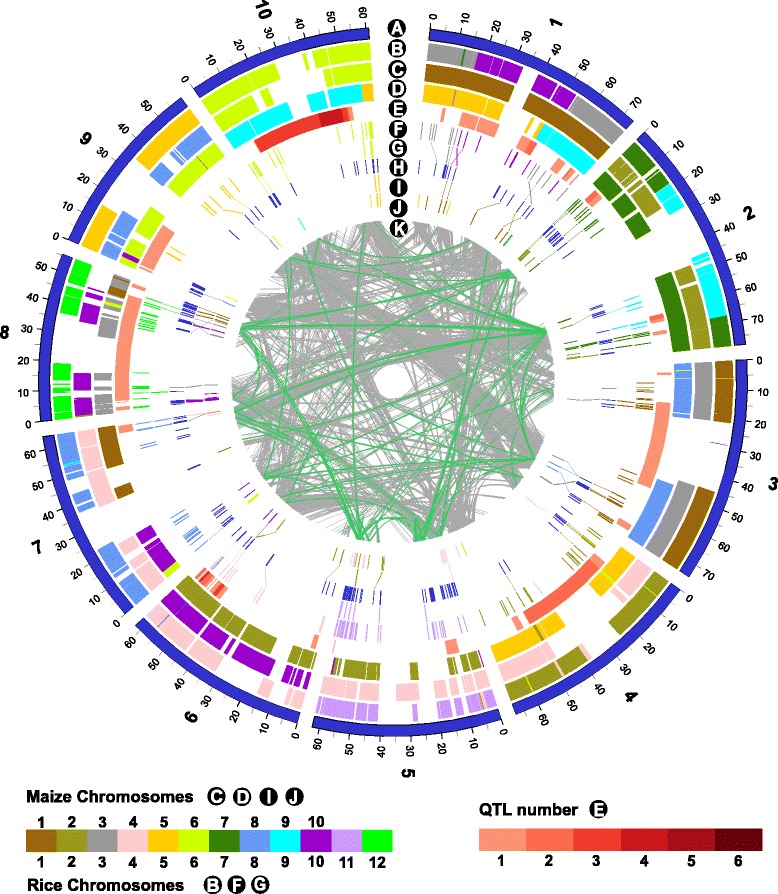
**Comparison of genome-wide distribution of NBS-encoding genes in** ***S. bicolor*** **,** ***Z. mays*** **and** ***O. sativa*** **.** Lanes detailed as follows; A: sorghum chromosomes, B: rice chromosomes, C, D: maize chromosomes, E: sorghum disease resistance QTL, F: rice NBS genes, G: homology between rice and sorghum NBS genes, H: sorghum NBS genes, I: homology between maize and sorghum NBS genes, J: maize NBS genes, K: duplicated genes in sorghum with NBS genes highlighted in green.

A total of 346 NBS-encoding genes, with highly conserved NBS regions, were identified within the ~700 Mb sorghum genome, accounting for ~ 1.2% of all predicted gene models in the sorghum reference genome [13] (Additional file 1: Table S1), comparable to the recent study in sorghum [14] which identified 348 NBS-encoding genes. Based on sequence similarity in the N-terminal and LRR domains, the 346 genes could be classified into 14 different NBS types (Table 1); of which 228 had LRR domains. The NBS-encoding genes were distributed unevenly across the genome (Figure 1), with over 60% located on 3 chromosomes (SBI-02, SBI-05 and SBI-08). Additionally, over two-thirds of the NBS-encoding genes (68.7%) were located in clusters on the chromosomes (Additional file 2: Table S2). The NBS-encoding genes were significantly enriched in the regions of the genome containing fungal pathogen disease resistance QTL (Χ^2^*p-*value 0.00272). Additionally, NBS-encoding genes were significantly enriched in regions of the genome identified as being under purifying selection. This pattern was observed both through domestication (Χ^2^*p-*value 0.000539) and improvement (Χ^2^*p-*value 0.0000046), characterized by elevated differentiation between wild, landrace and improved groups and with low nucleotide diversity and negatively skewed allele frequency spectra [23]. NBS-encoding genes were also enriched in chromosome regions under balancing selection (Χ^2^*p-*value 0.0323), with contrasting diversity and differentiation signatures to purifying selection. As a comparison, the distribution of the sorghum genes homologous to a set of 176 house-keeping genes identified in arabidopsis [24] were analysed and found not to be significantly enriched in the regions of the genome identified as being under purifying or balancing selection (Χ^2^*p-*value 0.956 and 0.202, respectively) or in the regions of the genome containing fungal pathogen disease resistance QTL (Χ^2^*p-*value 0.079).

**Table 1:** **The number of NBS-encoding genes per genome**

**Predicted protein domains**	**Letter code**	***S. bicolor***	***Z. mays***	***O. sativa***
NBS-LRR type genes				
NBS-LRR	NL	133	26	113
CC-NBS-LRR	CNL	24	7	39
NBS-LRR-CC	NLC	1	1	2
NBS-LRR-X	NLX	12	1	2
NBS-X-LRR	NXL	43	0	3
CC-NBS-LRR-X	CNLX	3	2	1
CC-NBS-X-LRR	CNXL	12	0	0
TIR-NBS	TN	0	0	1
Total		228	37	161
NBS type genes				
NBS	N	64	69	252
CC-NBS	CN	11	21	72
NBS-CC	NC	1	0	7
CC-NBS-X	CNX	3	0	2
NBS-CC-X	NCX	4	0	0
X-CC-NBS	XCN	8	0	1
X-NBS	XN	27	10	8
Total		118	100	342
Grand total		346	137	503

**Figure 2:**
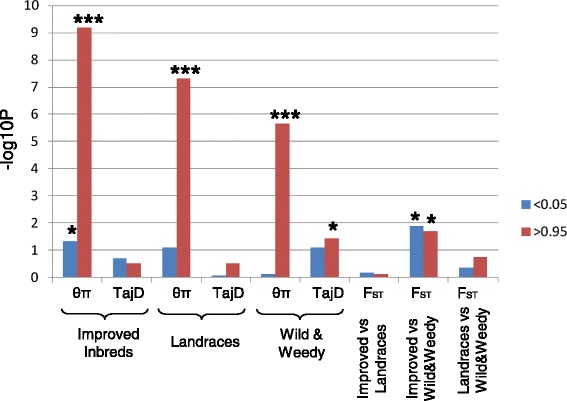
**Enrichment of sorghum NBS genes in the upper and lower 5% tail of the empirical distribution of 3 different population statistics (Pi, TajD (Tajima’s D).**

Polymorphism patterns in the NBS-encoding genes were also distributed unevenly across the genome. Overall, NBS-encoding genes had significantly higher diversity (*P* <3.18^e-9^ by paired t-test) in comparison to 346 randomly selected non NBS-encoding genes in the sorghum filtered gene set. NBS-encoding genes were significantly enriched in the upper 5% tail of the empirical distribution of the nucleotide diversity measure (θπ) for all genes in the sorghum filtered gene set (n = 29,346). This pattern was observed for all three groups (Χ^2^*p-*value <0.0001) (Figure 2); wild and weedy, landraces and improved inbreds. Differences between genotype groups were also observed in the nucleotide diversity levels in the NBS-encoding genes (Additional file 2: Figure S1) with diversity in the cultivated groups being consistently lower in comparison to the wild and weedy genotypes, an observation made previously genome-wide in both genic and non-genic regions [23]. A significant reduction, relative to all genotype groups, in NBS gene diversity, was only observed in the improved inbred group of sorghum genotypes (enrichment in the lower 5% tail of the empirical distribution of θπ; Χ^2^*p-*value 0.046) (Figure 2).

In total, just under 20% of all the NBS-encoding genes had patterns of molecular variation consistent with the action of selection, as measured by skewed diversity (θπ and θw), allele frequency spectra (Tajima’s D) and between group differentiation (FST) values. Just over half (38) of these NBS-encoding genes had signatures of purifying selection, with a drive towards beneficial allele fixation and selective removal of deleterious alleles through both natural and human-mediated selection. Eleven NBS-encoding genes were completely invariant in both the cultivated and wild groups, with a further 10 genes fixed only in the cultivated groups (Additional file 1: Table S1). The majority of these invariant genes (86%) occurred in gene clusters. The mlHKA test was used to validate whether the NBS-encoding domestication and improvement candidates showed patterns of genetic variation consistent with positive selection [25]. A model of directional selection best explained the patterns of polymorphism to divergence of the 17 variant candidate genes for domestication and improvement relative to 38 neutral loci (mean log likelihood ratio test statistic = 372; *P* < 0.0001 for all comparisons; Additional file 2: Table S3). In contrast, 23 NBS-encoding genes had molecular signatures consistent with balancing selection, in which multiple alleles were maintained at intermediate frequencies in ancestral and descendant populations. The mlHKA test identified that a model of balancing selection best explained the patterns of genetic variation in these 23 NBS-encoding domestication and improvement candidates (mean log likelihood ratio test statistic = 493.2; *P* < 0.0001 for all comparisons; Additional file 2: Table S3). Over 90% of the 23 NBS-encoding genes with signatures of balancing selection had an LRR domain, in contrast to only 52% of the 38 NBS-encoding genes with signatures of purifying selection (Additional file 2: Figure S2). Overall, NBS genes with LRR domains were more diverse than NBS-encoding genes without LRR domains, both in cultivated (θπ = 0.00278 with LRR domains vs θπ = 0.002203 without LRR domains) and wild and weedy groups (θπ = 0.00384 with LRR domains vs θπ = 0.00364 without LRR domains). Diversity also increased with increasing numbers of LRR domains across the three groups (Additional file 2: Figure S3).

NBS-encoding genes were more diverse in both regions under purifying and balancing selection (Table 2), in contrast to non-NBS encoding genes with signatures of selection, providing evidence of diversity in the NBS-encoding genes increasing subsequent to selection. The degree of diversity recovery subsequent to selection also varied according to the type of NBS-encoding gene, with the N class of NBS-encoding gene showing the largest amount of diversity recovery in the improved inbred group, the CNL class showing the largest amount of diversity recovery in the landrace group, and the XN class showing the largest amount of diversity recovery in the wild and weedy group (Additional file 2: Figure S4). Although in the minority, there were examples of NBS-encoding genes having lower diversity than non-NBS-encoding genes in regions under purifying selection. In all cases the NBS-encoding genes with lower diversity than non-NBS-encoding genes in regions under selection co-located with fungal pathogen biotic stress QTL, in particular anthracnose and rust resistance (Additional file 2: Figures S5-6).

**Table 2:** **The nucleotide diversity (θπ) in NBS-encoding genes in comparison to non-NBS-encoding genes across cultivated and wild and weedy sorghum genotype groups in regions of the genome under balancing and purifying selection**

**Selection type**	**Gene type**	**Cultivated**	**Wild and weedy**
Balancing	NBS-encoding gene	0.00675	0.00761
Balancing	Non NBS-encoding gene	0.00528	0.00499
Purifying	NBS-encoding gene	0.00023	0.00222
Purifying	Non NBS-encoding gene	0.00018	0.00169

The NBS-encoding genes identified with signatures of balancing selection had higher numbers of protein variants versus NBS-encoding genes under purifying selection (7.6 versus 4.3, respectively), in line with expectations of maintenance of an excess of polymorphism under balancing selection. Additionally the Ka:Ks ratio test, which compares the number of non-synonymous substitutions (potentially adaptive amino acid replacement changes) with the number of synonymous substitutions (assumed to be evolving neutrally), provided further evidence of adaptive substitutions accumulating at higher frequencies in the NBS-encoding genes with signatures of balancing selection in contrast to NBS-encoding genes with signatures of purifying selection (Table 3), likely due to the more frequent occurrence of advantageous nonsynonymous mutations in comparison to neutral sites. In comparison, the Ka:Ks ratio values were consistently lower for non-NBS encoding genes throughout the genome, whether under selection or neutral.

**Table 3:** **The Ka:Ks ratio for NBS-encoding genes and non NBS-encoding genes in regions under selection versus regions not under selection**

	**Under purifying selection**	**Under balancing selection**	**Not under selection**
NBS-encoding gene	0.459	0.705	0.653
Non NBS-encoding gene	0.122	0.318	0.347

Across all 346 NBS-encoding genes, the occurrence of non-functional alleles, either through frame-shifts or large effect SNPs (premature stop codons, start codon to non-start codon, stop codon to non-stop, splice site), ranged from 2.17% to 86.95% across the sorghum genotypes, with the wild and weedy genotypes having higher frequencies in comparison to the cultivated groups (Additional file 2: Table S4).

The NBS-encoding genes identified with signatures of balancing selection also had higher overall proportions of non-functional alleles, in comparison to NBS-encoding genes evolving under purifying selection or neutral expectations (Additional file 2: Figures S7). In the majority of cases (19/23 genes under balancing selection), less than 50% of the genotypes were impacted by frame-shifts or large effect SNPs, with multiple functional protein variants still present. Non-functional alleles occurred very rarely in NBS-encoding genes under purifying selection. Four of the NBS-encoding genes in sorghum (~1.2%) appeared to be psuedogenes in the sense that all of the alleles were rendered non-functional by frame-shifts or large deletions (>50% of gene). One of these genes (Sb05g007560; NXL classification) also had a signature of balancing selection and was in the upper 1% tail of the empirical distribution of the nucleotide diversity measure (θπ) for all genes in the sorghum filtered gene set, across cultivated and wild sorghum genotypes. This gene did not co-locate with a previously identified fungal pathogen biotic stress resistance QTL; however, it was observed that a differentiation between types of fungal pathogen biotic stress resistance QTL co-locating with NBS-encoding genes under purifying or balancing selection occurred. Almost 80% of QTL co-locating with NBS-encoding genes under purifying selection were associated with rust or anthracnose resistance. In contrast, over 80% of QTL co-locating with NBS-encoding genes with signatures of balancing selection were associated with ergot resistance, whereas only 1 QTL for rust and anthracnose did so.

To investigate signatures of selective sweeps, the genetic diversity of the genes in the 100 kb region flanking the NBS-encoding genes were analysed and found to be significantly reduced in comparison to genome-wide averages; with flanking genes enriched in the lower 5% tail of nucleotide diversity for all three groups (Additional file 2: Figures S8). In total, sixty-nine NBS-encoding genes were located within 100 kb of previously identified candidate genes under selection in sorghum [23], including previously described domestication genes, e.g. LA1, involved in tiller angle [26] and Rd, involved in pericarp colour [27].

### Polymorphism patterns in orthologous, paralogous and novel NBS-encoding genes in sorghum

**Figure 3:**
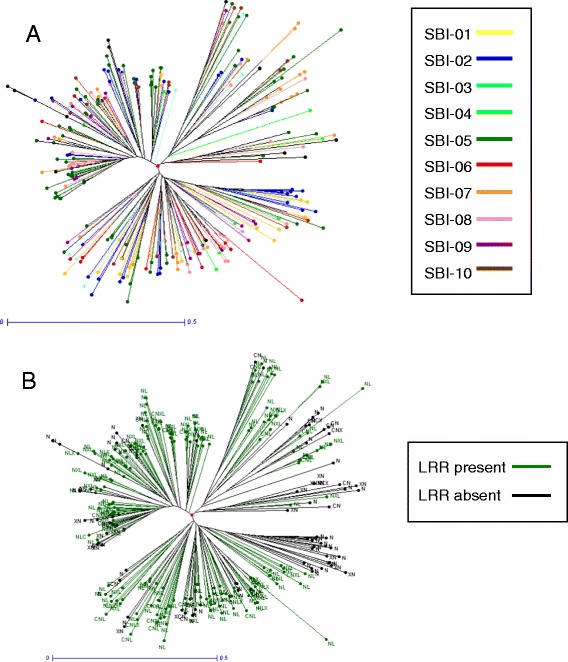
**Phylogenetic trees of NBS-encoding genes from sorghum. A**. Colour-coded by chromosome as indicated. **B**. Colour coded by presence or absence of LRR domain, as indicated, with gene letter codes as detailed in Table 1.

Phylogenetic trees, constructed for the NBS-encoding genes within and across sorghum, maize and rice species (Figure 3 and Additional file 2: Figures S9-11), allowed the identification of cross-species (orthologous) gene families. With a clade definition of nucleotide similarity <70% between clades (the genes in a clade identified as a multi-gene family), 647 clades were identified; 137 being multi-gene family clades containing 404 NBS-encoding genes in total; 85 of which belonged to 20 ancestral gene families, pre-dating species divergence (Additional file 2: Figure S12). The highest proportion of ancestral genes was identified in maize (21.1%), followed by sorghum (8.8%) and rice (6.3%). Ancestral gene families occurred predominately in gene clusters syntenic across species (67.1%). Overall, NBS-encoding genes in sorghum with signatures of selection (purifying or balancing) were more likely to be orthologous than neutral NBS-encoding genes, not under selection (Additional file 2: Figure S13). NBS-encoding genes with signatures of balancing selection in sorghum had the highest proportion of ancestral gene family members in comparison to NBS genes that were neutral or under purifying selection.

A more detailed phylogenetic analysis of sorghum gene families, specifically, indicated that at 70% nucleotide similarity and gene coverage, approximately one third of all NBS-encoding genes in sorghum belonged to paralogous, multi-gene families (Additional file 2: Figure S14). The majority (76%) of the paralogous genes were located within the same gene clusters, although over 12% of the gene families had paralogues located across multiple chromosomes (Additional file 2: Figure S15). 55% of paralogous NBS-encoding genes in sorghum also occurred within the recently duplicated super-gene regions of the genome, defined by 4DTv < 0.497 [23]. Overall, higher diversity levels were observed in paralogous NBS-encoding genes (θπ = 0.0039) in comparison to non-paralogous, singleton genes (0.0028; Additional file 2: Figure S16) and the proportion of paralogous NBS-encoding genes under selection was double that of singleton NBS-encoding genes in sorghum. Furthermore, the Ka:Ks ratio test identified a higher number of non-synonymous SNPs in paralogous NBS-encoding genes than in singleton genes (Additional file 2: Figure S17).

**Figure 4:**
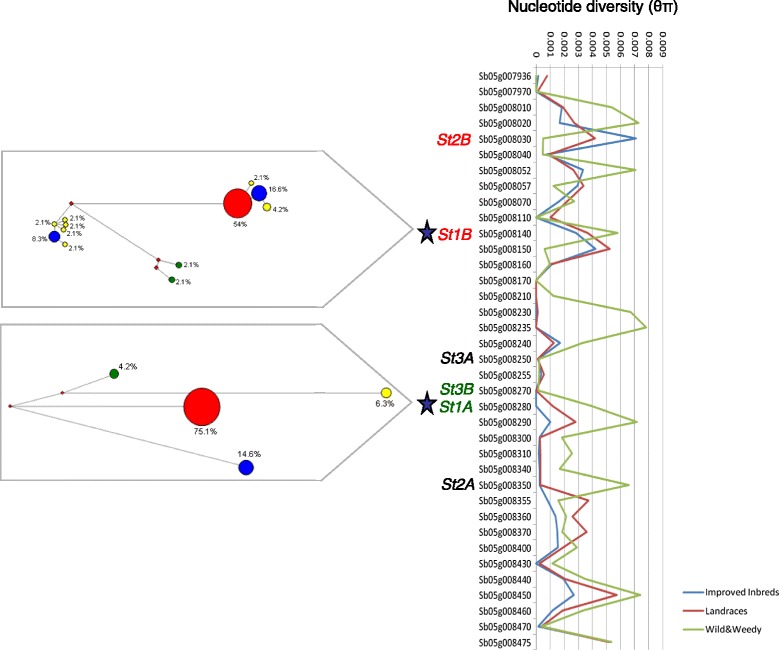
**Nucleotide diversity in NBS-encoding gene cluster on SBI-05.** The gene cluster is syntenous with gene clusters in rice (chr 11: Os11g11950, Os11g11960, Os11g11990) and maize (chr 4: GRMZM2G005347, GRMZM2G005452), with 6 candidate gene pairs for resistance to *Setosphaeria turcica* [28] highlighted and colour coded (red text: under balancing selection, green text: under purifying selection, black text: not under selection). Sorghum NBS-encoding genes belonging to the ancestral gene family also identified as being under purifying selection in rice [21] are highlighted with a blue star. Median-joining network trees are detailed for the ancestral gene pair St1B and St1A. Red nodes represent high frequency haplotypes. Blue nodes represent intermediate frequency haplotypes. Yellow nodes represent low frequency haplotypes. Green nodes represent *S. propinquum.*

Syntenic selective sweeps across species were identified through comparisons of NBS-encoding genes under selection in rice [21] and maize [22] from previous studies. In total, from 1508 improvement candidate genes and 1764 domestication candidate genes identified from the analysis of resequencing data of 75 wild, landrace and improved maize lines [22], six maize NBS-encoding genes were identified as being under purifying selection (5 through improvement and 1 through domestication). These maize NBS-encoding genes under selection were orthologous with 5 sorghum NBS-encoding genes also identified as being under purifying selection, including the candidate gene for anthracnose resistance *SbCg1* on SBI-05. In a similar recent study in rice [21], which analysed resequencing data of 40 cultivated and 10 wild progenitors, 2506 candidate genes under artificial selection were identified, eight of which were NBS-encoding genes. The rice NBS-encoding genes under selection were all located on chromosome 11 in 3 gene clusters, syntenous to three gene clusters on sorghum’s SBI-05 including gene clusters containing *SbCg1* and candidate genes against *Setosphaeria turcica*, the causal agent of northern leaf blight disease in maize [28]. The sorghum gene pair *St1A* and *St1B* identified [28] belonged to one of the 20 ancestral gene families identified across sorghum, maize and rice, including the maize orthologue which was found to have upregulated transcripts after fungal challenge of *Setosphaeria turcica.* While the orthologous maize gene was not identified as being under selection [22], the rice and sorghum orthologues in this ancestral gene family have been previously identified as being under selection. In sorghum, two contrasting signatures of selection occur in the *St1A* and *St1B* gene pair, with *St1A* (Sb05g008280) having a signature of purifying selection and *St1B* (Sb05g008140) having a signature of balancing selection (Figure 4). A second ancestral gene family was also identified as being under selection across species, containing the maize Rp1-D gene (GRMZM5G879178), sorghum genes Rp1-dp3 (Sb03g036450) and rph1-3 (Sb08g002410) for rust resistance, and the rice Pi37 (Os01g57310) gene for resistance to rice blast disease. This was recently identified as a rapidly evolving gene family, termed “Rp1/Pi37” [17], which had an effector response that confers resistance to multiple pathogens across species.

The novel genes identified previously in sorghum [23] were enriched for NBS-encoding genes (Χ^2^*p-*value 0.0051). In total, ten novel NBS-encoding genes were identified; all without an LRR domain (eight of which are classified as N, two as CN; Additional file 2: Table S5). Four novel genes were not present in any cultivated lines (Additional file 2: Figure S18); one was present only in *S.propinquum*, two only in the wild and weedy group and one in both *S. propinquum* and the wild and weedy group. Of the six novel genes that were present in at least one of the cultivated groups, one (novel_seq4_GLEAN_10000166) was present at high frequencies in all genotype groups except *S. propinquum*. All of the novel genes identified were in sorghum-specific gene families only.

## Discussion

This study, focusing on the most prevalent and ancient of the disease resistance gene families, has presented new data demonstrating that NBS-encoding genes are under strong selection pressure in sorghum, through the contrasting evolutionary processes of purifying and balancing selection, and that they are enriched in the regions of the genome associated with fungal pathogen disease resistance. This study has also observed that NBS-encoding genes ancestral to cereals were less diverse than sorghum lineage-specific NBS-encoding genes and that the ancestral genes were more abundant in regions with signatures of selection (purifying or balancing) than in regions not under selection. This knowledge of the variation patterns of the different types of disease resistance genes indicates that NBS-encoding gene family members have real value for agriculture and can provide plant breeders with new tools to more effectively develop enhanced crop varieties with more durable resistance to plant fungal pathogens.

### NBS-encoding genes have a role in the domestication of sorghum through contrasting mechanisms

The domestication syndrome is traditionally associated with traits such as tillering and seed shattering, with only limited studies to date supporting its association with disease resistance (e.g. [29]). The current study found that regions of the genome under purifying and balancing selection through both domestication and improvement were enriched for NBS-encoding genes. Two-thirds of the NBS-encoding genes under selection were associated with domestication rather than improvement which may have been influenced by the perennial nature of the wild relatives of sorghum, in contrast to the annual life-cycle of the majority of cultivated types, through increased plant longevity in the face of constant selection pressure for disease resistance. There was also evidence of NBS-encoding genes recovering and maintaining diversity more rapidly than non-NBS-genes in these regions. Only a few exceptions were observed, notably in NBS-encoding genes co-locating with QTL for resistance to anthracnose, which could reflect the variable selection pressures imposed by different pathogens. Additionally, an analysis of genomic regions under selection in a set of 539 advanced sorghum genotypes as part of the preliminary yield trials of the Australian sorghum pre-breeding program, based in Queensland, identified that the majority (58.3%) of genomic regions under selection co-located with NBS-encoding genes (data not shown).

Despite ~12% of the NBS-encoding genes in sorghum having signatures of purifying selection, with concordant reduction in diversity, overall NBS-encoding genes had three times more nucleotide diversity than non-NBS-encoding genes (θπ = 0.0031 versus θπ =0.0018, respectively) with an additional ~8% showing specific signatures of balancing selection. Such heterogeneity of the impact of selection on NBS-encoding genes has been previously reported [5] with the NBS domain being more commonly subject to purifying selection, in contrast to the more variable LRR domain. This likely reflects the role of the LRR domain in recognition of constantly evolving pathogen ligands and a role for the NBS domain in recognition signalling [30]. The results of the current study are in-line with this finding and have demonstrated that while over 90% of the NBS-encoding genes with a signature of balancing selection had an LRR domain, this was reduced to only 50% in the NBS-encoding genes under purifying selection. The observed increase in nucleotide diversity with a concomitant increase in the number of LRR domains per gene emphasizes the highly variable nature of the LRR proteins.

### An integrated fungal pathogen disease-QTL map and NBS-encoding genes reveals regions of the sorghum genome associated with multiple quantitative disease resistance traits

Regions of the genome containing fungal pathogen related biotic stress resistance QTL were found to be significantly enriched for NBS-encoding genes. We also found that the diversity of the NBS-encoding genes can vary according to the particular type of co-locating biotic stress resistance QTL, e.g. NBS-encoding genes underlying anthracnose QTL were significantly less diverse than the mean diversity of NBS-encoding genes in cultivated sorghum (θπ = 0.0007 versus θπ = 0.0026, respectively).

Previous studies investigating the coincidence of NBS-encoding genes and disease resistance QTL in other species including rice [31] and soybean [32] also found a significant enrichment of the NBS-encoding genes in the QTL fraction of the genome. Approximately 36% of the sorghum genome was implicated in quantitative disease response (QDR) to fungal pathogens, less than reported recently for rice, 54% [31]. Furthermore, nearly half of the total QDR genomic space consisted of co-localising QTL for the same trait, indicating the robustness of the disease resistance QTL identified. The majority (>90%) of co-localising QTL conditioned resistance to multiple diseases, with a stand-out hotspot region on the long arm of SBI-06, containing 15 QTL for 6 traits from 4 studies [33-36]. Such hotspots for multiple disease resistances have been noted in other species, including maize [37], rice [31], and potato [38]. It is thought this could be due to single gene effects, whereby the resistance gene and QTL are allelic, or by the effects of clusters of genes. There were 7 NBS-encoding genes in the disease QTL hot-spot region on SBI-06; 2 singletons and 1 cluster of 5 genes, indicating that either individual genes can provide resistance against multiple pathogens or alternative resistance mechanisms are involved. Members of the defense-associated transcriptor family WRKY (n = 69) and MYB genes (n = 110) were investigated for the correspondence with fungal pathogen related biotic stress QTL in sorghum, however these genes were not found to be significantly enriched (Χ^2^*p-*value 0.21 and 0.052, respectively) in the QDR genomic space. Moreover the distribution of the sorghum genes homologous to a set of 176 house-keeping genes identified in arabidopsis [24] was compared to the QDR genomic space and also found not to be significantly associated.

### Deleterious mutations and presence/absence variations contribute to the rapid variation in NBS-encoding genes in the grasses

Similar to previous findings [13,39,40], the number of NBS-encoding genes in rice (503) was almost 4 times higher than in maize (137) and one and a half times higher than sorghum (346; Table 1), with no TIR-encoding NBS genes identified in sorghum, rice or maize [39,41]. Temporal difference in NBS-encoding gene expansion among species was estimated by examining the proportion of multi-gene families across similarity/coverage thresholds (Additional file 2: Figure S19). In line with recent findings [15], considerably smaller proportions of NBS-encoding genes were found between 60% and 80% similarity in maize, in comparison to sorghum and rice, indicating that recent duplications were likely to have dominated the NBS-encoding genes in the maize genome with more ancient duplications observed in sorghum and rice. A high degree of clustering was also a significant feature of the NBS-encoding genes across the three genomes, with rice having proportionally more NBS-encoding genes in clusters (72.9%), potentially due to a higher number of localised tandem repeats, in comparison to sorghum (68.7%) and maize (51.4%). Of the defined gene clusters across genomes, maize was 100% syntenic across species, in contrast to sorghum (75.6%) and rice, which had the lowest proportion of syntenic gene clusters (58%) (Additional file 2: Figure S20). Rice had almost twice as many species-specific genes in clusters (72.4%) in comparison to sorghum (42.8%) and maize (43.4%) (Additional file 2: Figure S21). Interestingly, the NBS-encoding genes with signatures of balancing selection in sorghum were less likely to be located in clusters than NBS-encoding genes with signatures of purifying selection or those evolving neutrally (61%, versus 92% versus 70%, respectively), potentially indicating a fitness cost of NBS-encoding genes. The high proportion of NBS-encoding genes with signatures of purifying selection that were located in clusters provides further support for lineage specific rearrangements driving rapid evolution and the fixation of beneficial alleles. Such lineage-specific tandem duplications have led to higher numbers of gene copy variants in rice, in comparison to sorghum and maize, in line with the recent findings [40]. This highly dynamic clustering, through lineage specific rearrangements, is potentially a key mechanism driving the plasticity of NBS-encoding genes in the grasses via presence/absence variation (e.g. gene loss events, psuedogenization and novel genes) and copy number variation.

It has also been previously noted that the plasticity of disease resistance genes in plants can be mediated by gene regulators, including microRNAs (miRNA) [40]. miRNAs have been shown to play an important regulatory role in the growth and development of eukaryotes [42]. Specifically, they have been shown to regulate the expression of a number of key stress-related genes in plants (e.g. [43]). Amongst the cereals, sorghum and rice have higher percentages of NBS-encoding genes targeted by miRNA (37.5 and 36.4% respectively) in comparison to maize (28.94%) [40]. It is possible that such high proportions of miRNA targeting disease resistance genes may be associated with increased functional redundancy in rice and sorghum in contrast to maize, through higher proportions of species-specific NBS-encoding gene copies; 84.6% in rice, 71.3% in sorghum and 52.9% in maize. It has been speculated that miRNAs have a role as a dosage regulator, through expression-level repression following large or local genome duplication events [40].

Previous studies looking at the numbers of NBS-encoding genes across grass species have noted the rapid nucleotide evolution at the NBS-loci and the greater tendency for gene loss or gene number variation in contrast to house-keeping genes [15,39]. It has been postulated [15] that natural selection could be responsible for the drastic variation in numbers of NBS-encoding genes across rice, maize, sorghum and brachypodium, where the rapid expansion and/or contraction is a fundamentally important strategy for a species to adapt to a quickly changing spectrum of pathogens. The high frequency of NBS-encoding genes with non-functional alleles could indicate that there is a fitness cost associated with the disease resistance genes. For example, NBS-encoding genes that do not have useful functions in an environment lacking specific pathogens would more likely be lost or become pseudogenes through loss of function mutations to avoid a fitness cost. In the majority of cases the fixation of the null alleles was not observed, however in one case a null allele, caused by a premature stop SNP, in Sb08g005620 was found to be fixed across the cultivated genotypes (Additional file 2: Figure S22). This gene also had a signature of balancing selection however the fixation of the null allele in the cultivated lines could indicate the dual roles of both purifying selection to drive an increase in the frequency of new favourable mutations and balancing selection to maintain the different alleles.

It has been speculated previously [15] that multi-gene families facilitate the rapid evolution of NBS-encoding genes via frequent sequence exchange to generate novel gene sequences that may encode altered specificities. Our findings of increased polymorphism, and in particular increased non-synonymous polymorphisms (Ka:Ks ratio in cultivated sorghum of multi-gene families of 0.74 versus only 0.57 in the singleton genes in multi-gene families) support this hypothesis. The finding that the NBS-encoding genes from cross-species families were more abundant in regions under selection in sorghum supports previous results (e.g. [17]) that a diverse repertoire of NBS-encoding genes that provide resistance against rapidly evolving pathogens occurs not only within species but also across species.

### Complex evolutionary dynamics in the evolution of NBS-encoding genes identified across species

Although there have been numerous studies characterising NBS-encoding genes across species, to date there have been limited comparable studies characterising NBS-encoding gene polymorphisms within species. Existing studies also report higher nucleotide diversity in NBS-encoding genes in arabidopsis [16] and rice (e.g. [12,21,44]) in comparison to genome-wide values. A study in arabidopsis [16] focused specifically on the evolutionary dynamics of a subset of 27 NBS-encoding genes by sequencing the LRR domain in 96 *A. thaliana* accessions, and observed a continuum of possible states in the evolutionary processes, including selective sweeps and balancing selection with many stages in between. Although several loci could be identified as candidates for recent selective sweeps, they found that this scenario was not common and overall found limited evidence for selective sweeps and hence, did not support the co-evolutionary arms-race hypothesis as a general evolutionary model for this group of genes. Additionally, only weak evidence was found for signatures of balancing selection acting to maintain multiple alleles at intermediate frequencies over prolonged periods of time. However, a recent genome-wide resequencing study in rice [21], looking at a subset of 102 NBS-encoding genes, found some evidence to support selective sweeps in cultivated rice, with 23 NBS-encoding genes showing significantly lower diversity than the genome average. The dynamic nature of a larger set of 725 NBS-encoding genes in rice was also demonstrated in a recent study [44], which showed that presence/absence polymorphism, caused by frequent deletions and translocations, were prevalent between different accessions of rice, in addition to across grass species. Another study in rice [12] also identified a high frequency of presence/absence variation in a subset of NBS-encoding genes between 21 cultivated and 14 wild rice populations, and postulated that such variation could potentially result from geographic differentiation. Pathogen prevalence and virulence is undoubtedly impacted by geographic differentiation and hence such expansion and/or contraction in the number of NBS-encoding genes appears to be an important strategy for many cereal species to adapt to the quickly changing spectrum of pathogens. Segmental and tandem duplications and gene conversion are likely to have contributed to the high degree of clustering of NBS-encoding genes across species (e.g. [40]), further leading to synteny erosion and gene loss. The current study also identified presence/absence variation in ~5% of all the NBS-encoding genes in sorghum, with 10 ten novel NBS-encoding genes identified, in addition to 8 genes with gene-loss events observed. This demonstrates the on-going dynamic and highly plastic nature of the largest of the disease resistance gene families.

## Conclusions

A key mechanism driving the rapid variation in NBS-encoding genes in the cereals is the highly dynamic clustering, through lineage specific rearrangements via presence/absence variation (e.g. gene loss events, psuedogenization and novel genes) and copy number variation. Multiple evolutionary processes drive the plasticity of the NBS-encoding genes in sorghum; with the nucleotide sequence summary statistics depicting a continuum of possible states in the evolutionary process including both purifying and balancing selection. Such contrasting evolutionary processes have impacted ancestral genes, across the cereals, more than species-specific genes in sorghum.

As plant breeders seek to identify and deploy robust disease resistances, this study provides them with a clear understanding of the origins and allelic diversity of this rich gene family so important to the past, present and future of sorghum, a staple food crop for half a billion people. More broadly, by understanding the mechanisms that cereals use in the “arms race” with rapidly evolving fungal pathogens through NBS-encoding gene family expansion via duplication and rearrangements, researchers and breeders are better equipped to effectively manipulate the plants defenses in this continuing battle.

## Methods

### Identification of NBS-encoding genes

Genome assemblies and predicted gene models for sorghum (v1.4) [13], maize (v1.0) [19] and rice (*O. sativa* subsp. *japonica*; Release 7) [18,45] were obtained from JGI (http://www.phyotozome.net/x), Maizesequence.org (http://ftp.maizesequence.org/current/filtered-set/) and MSU (ftp://ftp.plantbiology.msu.edu/pub/data/Eukaryotic_Projects/o_sativa/annotation_dbs/pseudomolecules/version_7.0/all.dir/) respectively. To identify NBS-encoding genes in the three grass species, BLASTp with the amino acid sequence of the NB-ARC domain (Pfam: PF00931) was used; the threshold expectation value was filtered on 10–4. Subsequently the Pfam (Protein family) database was used to determine whether the corresponding candidate NBS protein encoded TIR, NBS or LRR motifs. Each of the candidate genes was subsequently checked manually by using existing annotations in GenBank to confirm that they encoded the expected NBS proteins. COILS under a threshold of 0.9 was then employed to specifically detect CC (coiled coils) domains. In each species genome, a gene cluster was defined if two or more NBS-encoding genes were located within 200 kb [46].

### Sequence alignment and phylogenetic analysis

Multiple alignments of the predicted amino acid sequences of the conserved NBS domain (PFAM00931) were performed by ClustalW [47] and Mega5.0 [48]. The extent of nucleotide divergence and gene coverage were calculated to identify gene families between all identified NBS-encoding genes both within each species and across all three species using a previously described perl script [4]. Phylogenetic trees were constructed using the aligned nucleotide sequences of the NBS protein domain (PFAM00931) in TreeBest based on the neighbour-joining method. The tree was displayed using DARwin5.0 software [49]. Syntenic regions of the whole genomes of sorghum, rice and maize [50], with the locations of the identified NBS-encoding genes highlighted, was displayed using the software Circos [51].

### Data Analysis

The sequence data of all of the identified NBS-encoding genes in sorghum were extracted from the whole genome resequencing data generated across 44 sorghum genotypes [23], representing three groups (wild and weedy group, landrace group and improved inbred group) (Additional file 3: Table S7). The following summary statistics were calculated as previously described: the average pairwise divergence within a group (θπ), the Watterson’s estimator (θw) and Tajima’s D were estimated for the identified NBS-encoding genes and the surrounding 10 kb genomic interval of the three groups were calculated using a BioPerl module and an in-house perl script. F_ST_ was calculated, based on the same windows, to measure population differentiation using another BioPerl module. Summary statistics involving coding regions included numbers of synonymous (Ks) and nonsynonymous (Ka) substitutions, were calculated utilising the KaKs_Calculator1.2 software (MYN method) [52], and the number of protein variants based on nonsynonymous substitutions.

Regions of the genome under purifying selection were previously identified using the population genetics summary statistics (θπ, θw, Tajima’s D and F_ST_) in the following three population pairwise comparisons: (i) wild and weedy versus landraces to identify domestication events, (ii) landraces versus improved inbreds to identify improvement events, (iii) wild and weedy versus improved inbreds to identify both domestication and improvement events. NBS-encoding genes with signatures of purifying selection were identified from the candidate genes previously described [23] in addition to the identification of NBS-encoding genes in the top and bottom 5% tails of the empirical distribution of the population summary statistics. The population genetics summary statistics were also used to identify signatures of balancing selection with the following criteria; θπ and θw in the upper 25% of the empirical distribution; Tajima’s D in the upper 5% of the empirical distribution and F_ST_ values <90% of the population pairwise distribution. Median-joining networks were constructed for selected NBS-encoding genes separately in Network [53]. A set of 17 of the non-invariant candidate genes under purifying selection were used as input, together with 38 neutral genes, for the mlHKA test for validation purposes [25]; in addition to a set of 23 candidate genes under balancing selection, together with the same set of 38 neutral genes. The mlHKA program was run under a neutral model, where numselectedloci = 0, and then under a selection model, where numselectedloci > 0. Significance was assessed by the mean log likelihood ratio test statistic, where twice the difference in log likelihood between the models is approximately chi-squared distributed with df equal to the difference in the number of parameters. Duplicated gene pairs were identified using the four-fold degenerate transversion (4DTv) ratio calculated previously [23].

Presence/absence patterns were identified across the 44 sorghum genotypes resequenced. BLASTp analysis with the amino acid sequence of the NB-ARC domain (Pfam: PF00931) was used for all 101 novel genes identified previously [23]; as previously the threshold expectation value was filtered on 10–4. Additionally the gene loss events were identified using read depth at 100 bp resolution from all identified NBS-encoding genes in across all genotypes, as described previously [23].

### An integrated disease-QTL map

26 sorghum fungal pathogen resistance QTL were retrieved from the set of 771 QTL projected onto the sorghum consensus map [54]. An additional 40 fungal pathogen disease resistance QTL and/or major effect genes from 4 additional studies [35,36,55,56] were also projected onto the sorghum consensus map following the same strategy. The physical locations of a total of 66 fungal pathogen disease resistance QTL representing 9 traits from 11 studies (Additional file 2: Table S6) were predicted using the framework map of 504 sequenced markers with known genetic linkage distances, as detailed previously [54].

### Availability of supporting data

The data sets supporting the results in this article are available from Dryad: doi:10.5061/dryad.d334b.

## Additional files

Additional file 1: Table S1. Detailing the location, type and selection status of 346 NBS-encoding genes identified in sorghum. Additional file 2:
**Contains 22 supplementary figures and 5 supplementary tables, all referenced in the main text.**
Additional file 3: Table S7. Detailing the sorghum genotypes included in the current study.

## Abbreviations

NBS-LRR: Nucleotide-binding site plus leucine-rich repeat
QTL: Quantitative trait loci
TIR: Toll/interleukin-1 receptor
QDR: Quantitative disease response
miRNA: microRNAs
