# Supplementary material for: The plasticity of NBS resistance genes in sorghum is driven by multiple evolutionary processes
Source: BMC Plant Biol. 2014 Sep 26;14:253. doi: 10.1186/s12870-014-0253-z (PMC4189741; doi:10.1186/s12870-014-0253-z)
Supplement: Additional file 2: — Contains 22 supplementary figures and 5 supplementary tables, all referenced in the main text. [file 12870_2014_253_MOESM2_ESM.pdf]

Additional File 2 for:

The plasticity of NBS resistance genes in sorghum is driven by multiple evolutionary processes

Mace ES, Tai SS, Innes DJ, Godwin ID, Hu WS, Campbell BC, Gilding EK, Cruickshank A, Prentis PJ, Wang J, Jordan DR

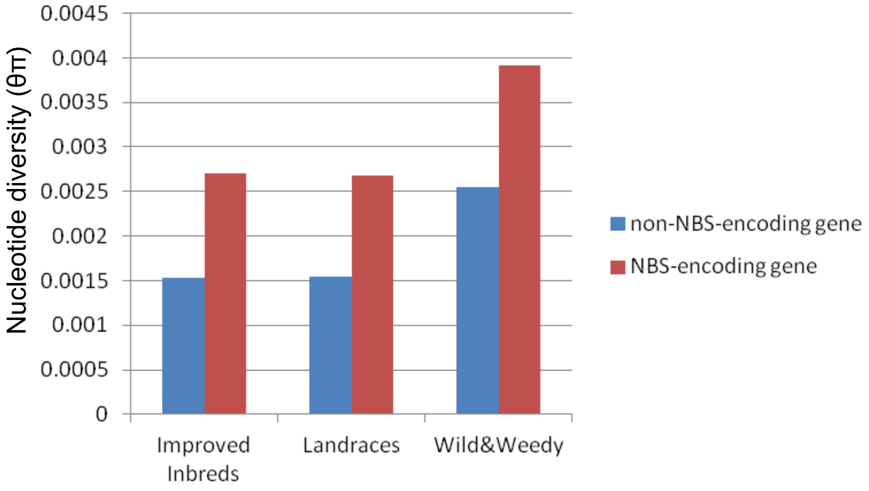

Figure S1. Nucleotide diversity (θπ) in NBS-encoding genes in comparison to non-NBS-encoding genes across the three sorghum groups.

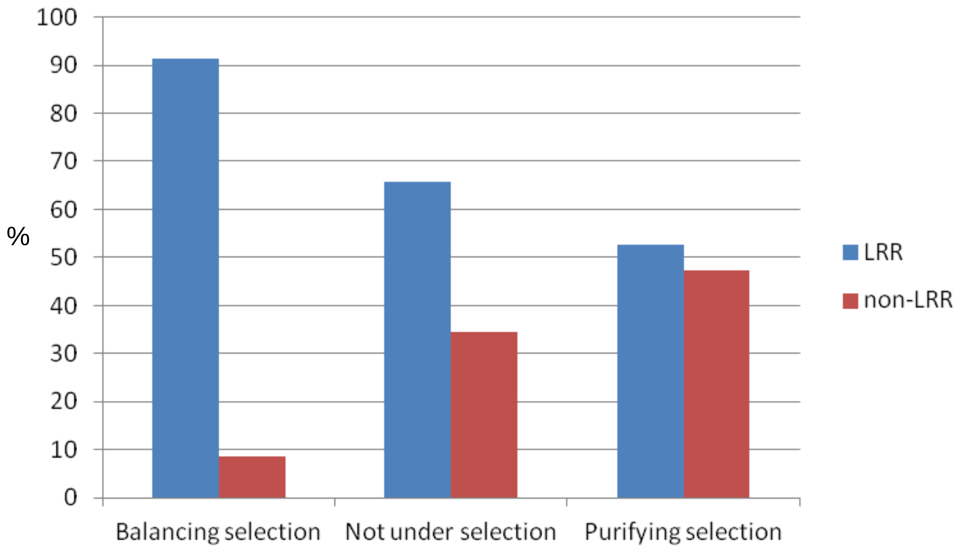

Figure S2. Proportion of NBS-encoding genes with LRR domains that are under balancing selection, not under selection or under purifying selection.

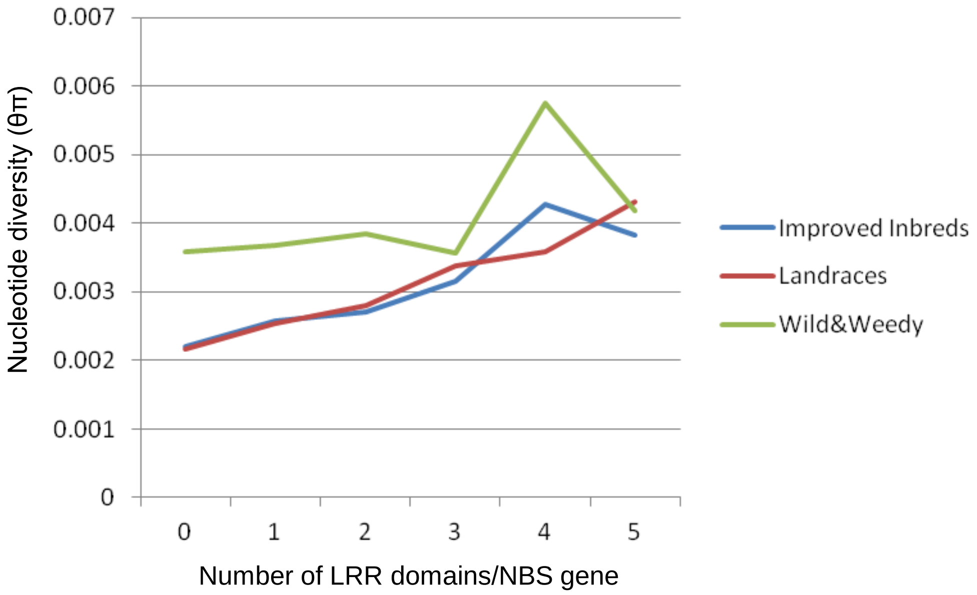

Figure S3. Diversity (θπ) of NBS-encoding genes with increasing numbers of LRR domains across sorghum groups

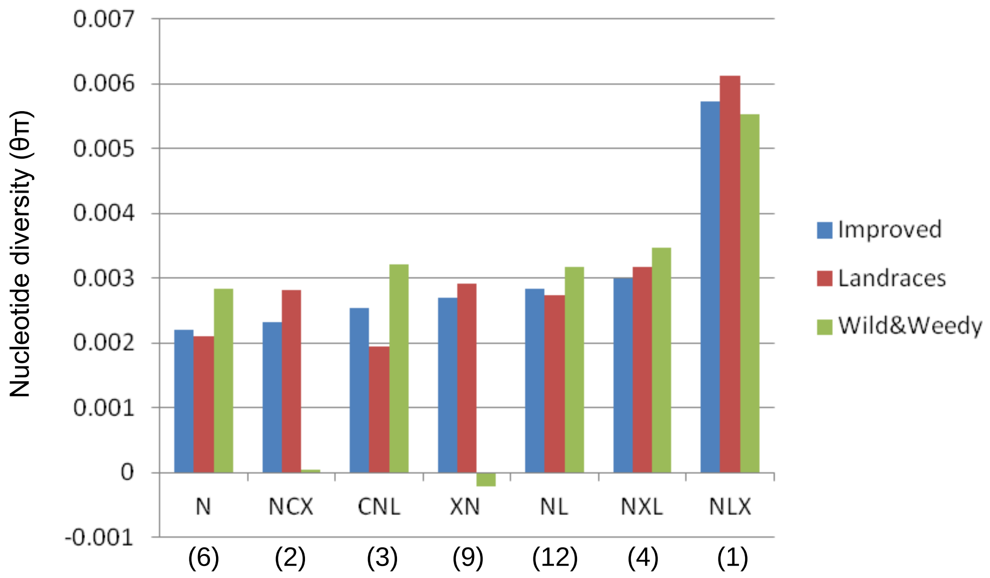

Figure S4. Difference in nucleotide diversity between genes under selection in comparison to genes not under selection in the different NBS-encoding gene types. The numbers in brackets indicate the total number of genes included in the comparison for each gene type.

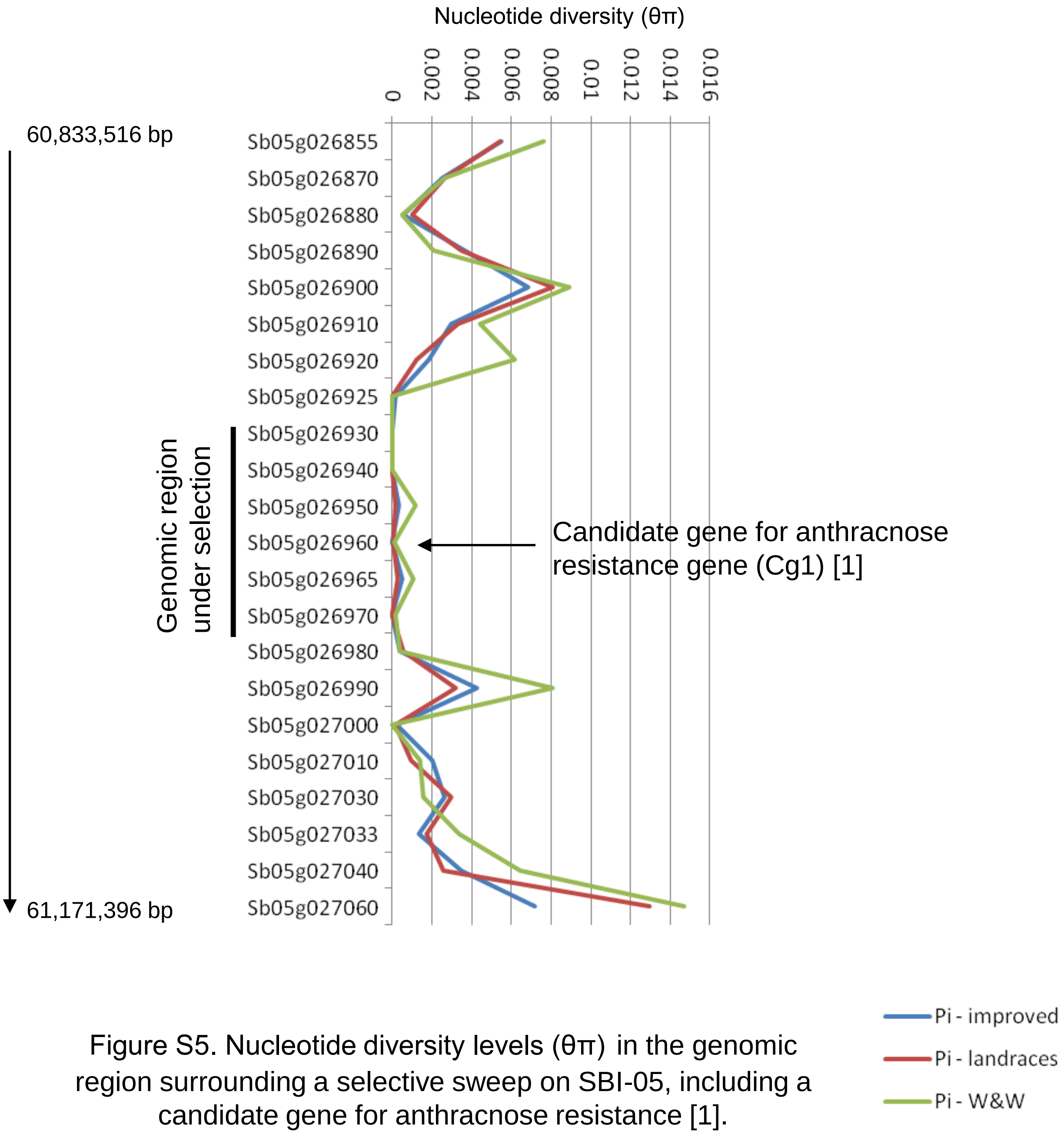

Figure S5. Nucleotide diversity levels (θπ) in the genomic region surrounding a selective sweep on SBI-05, including a candidate gene for anthracnose resistance [1].

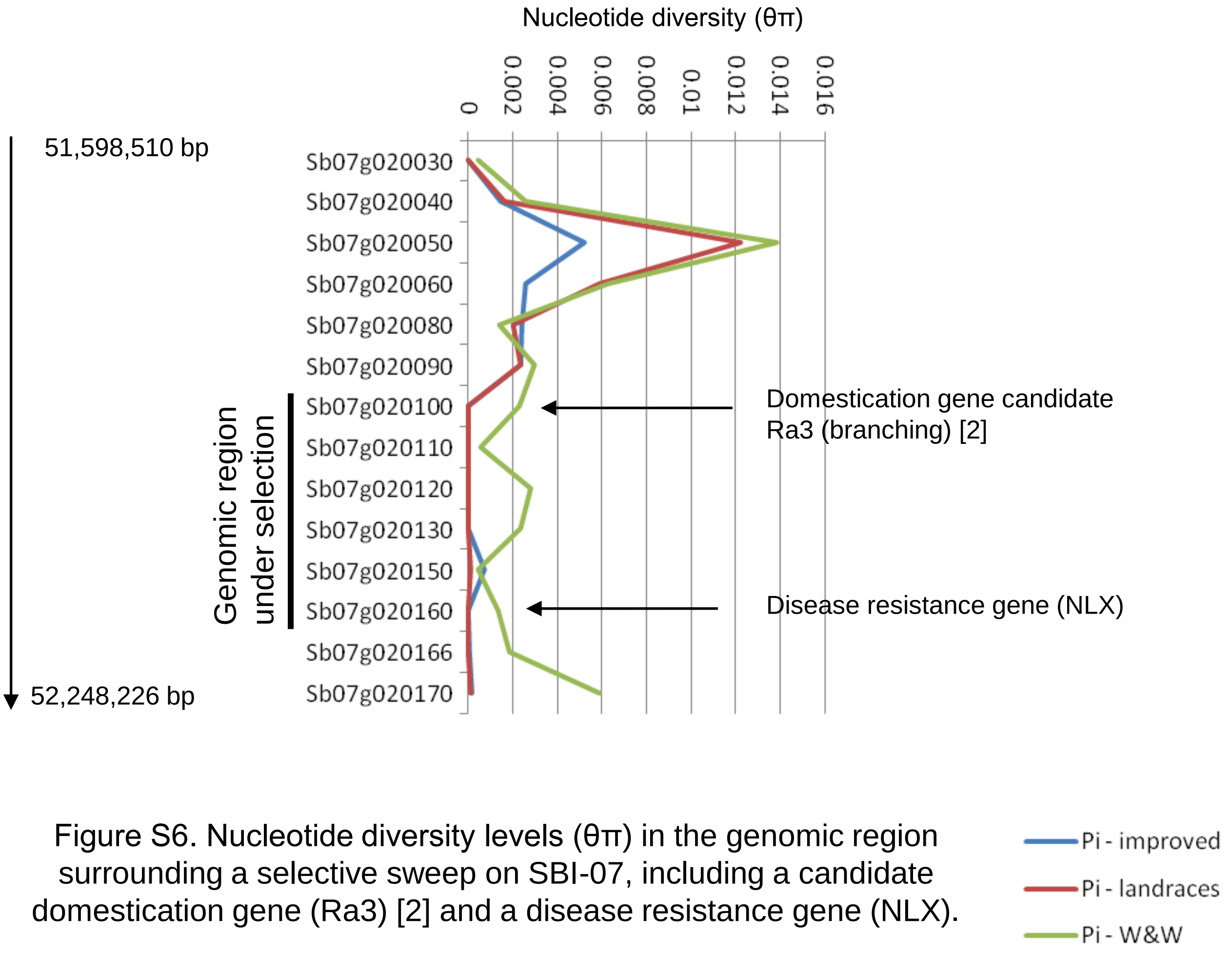

Figure S6. Nucleotide diversity levels (θπ) in the genomic region surrounding a selective sweep on SBI-07, including a candidate domestication gene (Ra3) [2] and a disease resistance gene (NLX).

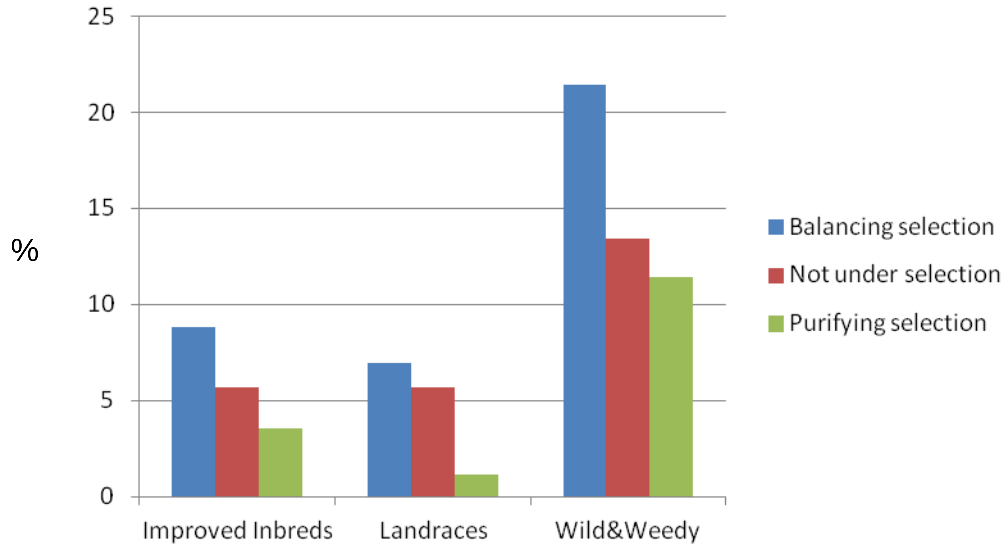

Figure S7. Proportion of NBS-encoding genes with large effect SNPs across groups and selection types

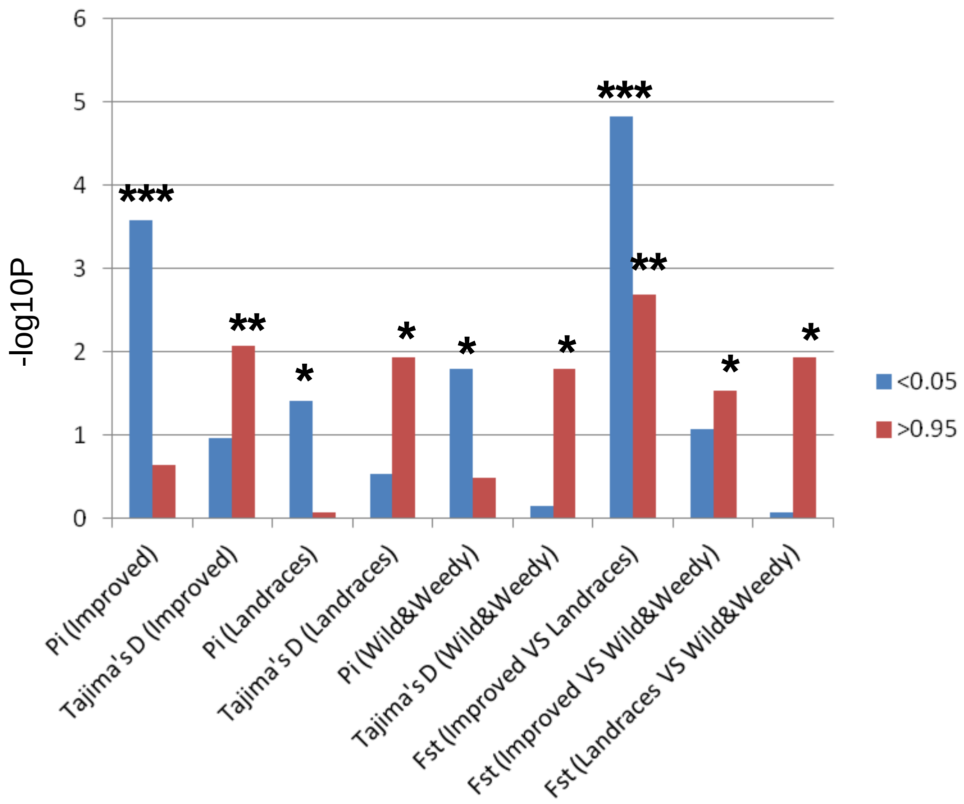

Figure S8. A summary of the results of a chi-square test to determine whether the number of genes **flanking NBS-encoding genes** (within 100kb) located in the upper and lower 5% tail of the empirical distribution of 3 different population statistics (pi: $\theta\pi$, Tajima's D and F_{ST}) differ significantly from that expected by chance

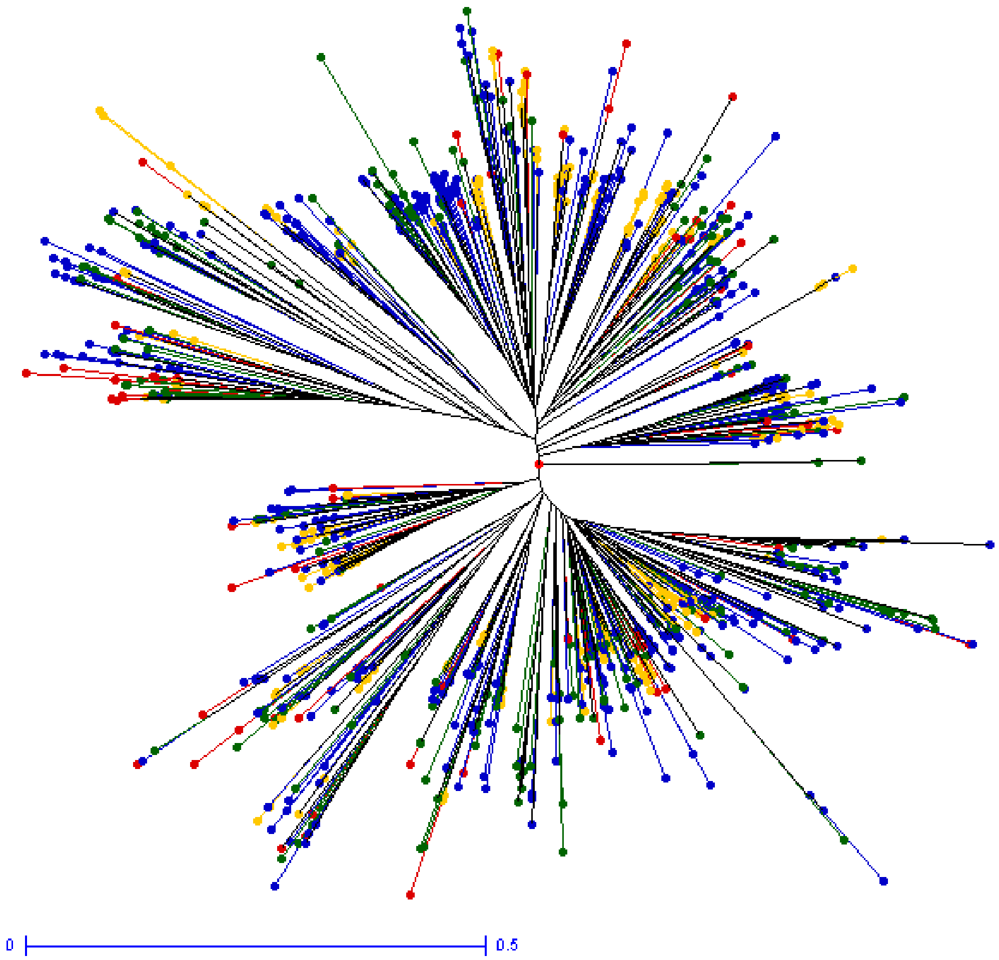

Figure S9. Phylogenetic relationships of NBS-encoding genes from sorghum, rice and maize; a simplified neighbor-joining tree colour-coded as follows: NBS-encoding genes specific to rice in blue, specific to sorghum in green and specific to maize in red; cross-species gene families in yellow.

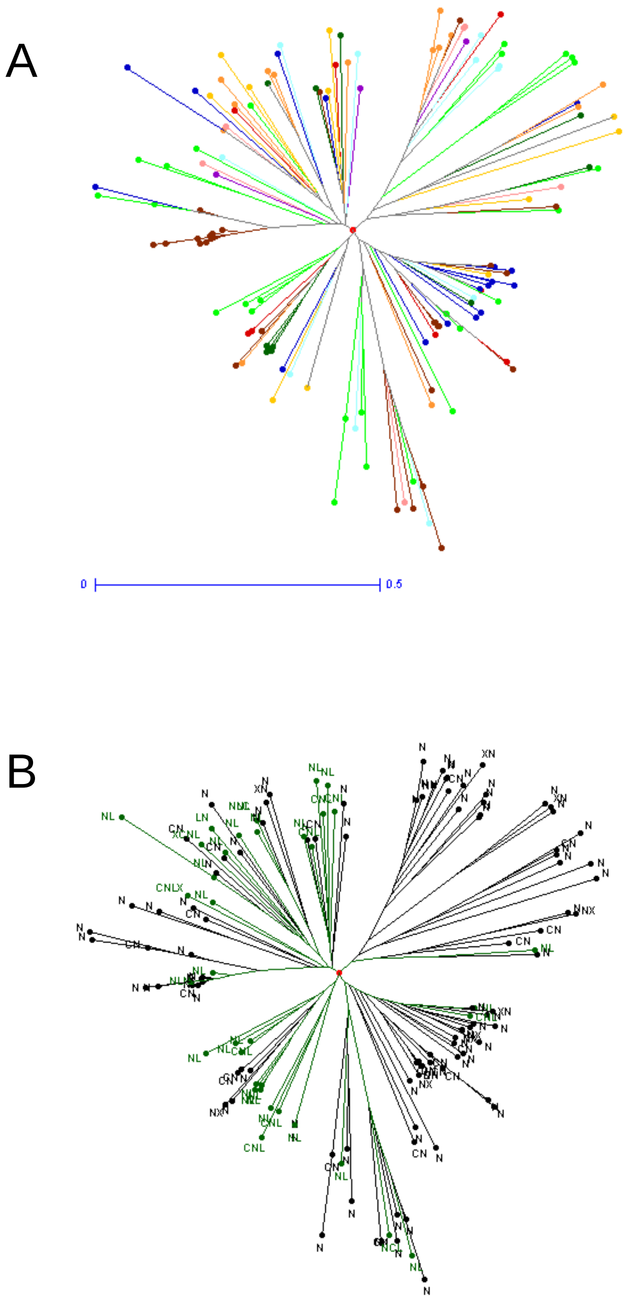

Figure S10. Phylogenetic trees of NBS-encoding genes from maize. A. Colour-coded by chromosome, chr 1: yellow, chr 2: dark blue, chr 3: light blue, chr 4: light green, chr 5: dark green, chr 6: red, chr 7: orange, chr 8: pink, chr 9: purple, chr 10: brown. B. Colour coded by presence or absence of LRR domain, green and black respectively, with gene letter codes indicated.

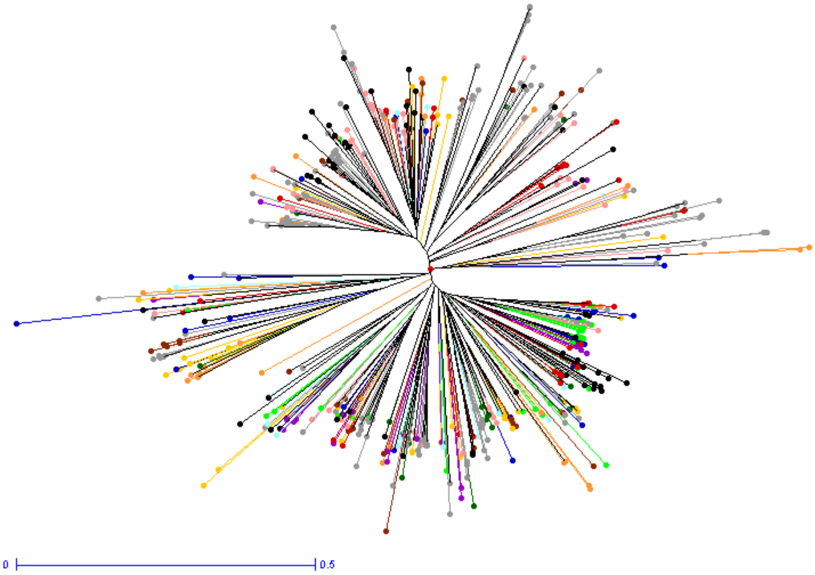

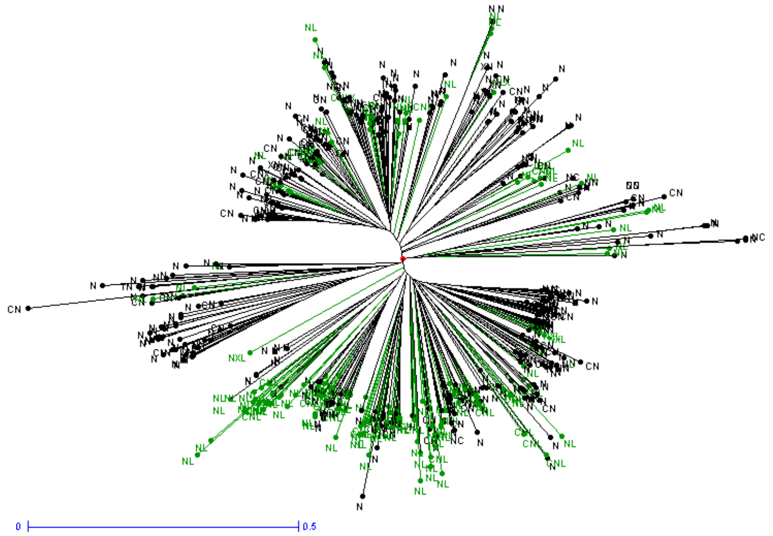

Figure S11. Phylogenetic trees of NBS-encoding genes from rice. A. Colour-coded by chromosome, chr 1: yellow, chr 2: dark blue, chr 3: light blue, chr 4: light green, chr 5: dark green, chr 6: red, chr 7: orange, chr 8: pink, chr 9: purple, chr 10: brown, chr 11: grey, chr 12: black. B. Colour coded by presence or absence of LRR domain, green and black respectively, with gene letter codes indicated.

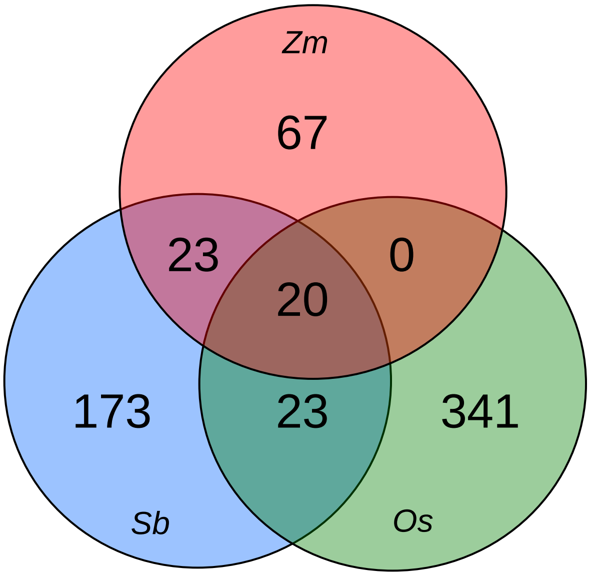

Figure S12. NBS-encoding gene families in common across species (*Zm*: *Zea mays*; *Sb*: *Sorghum bicolor*; *Os*: *Oryza sativa*) at a 70/70 similarity/coverage threshold

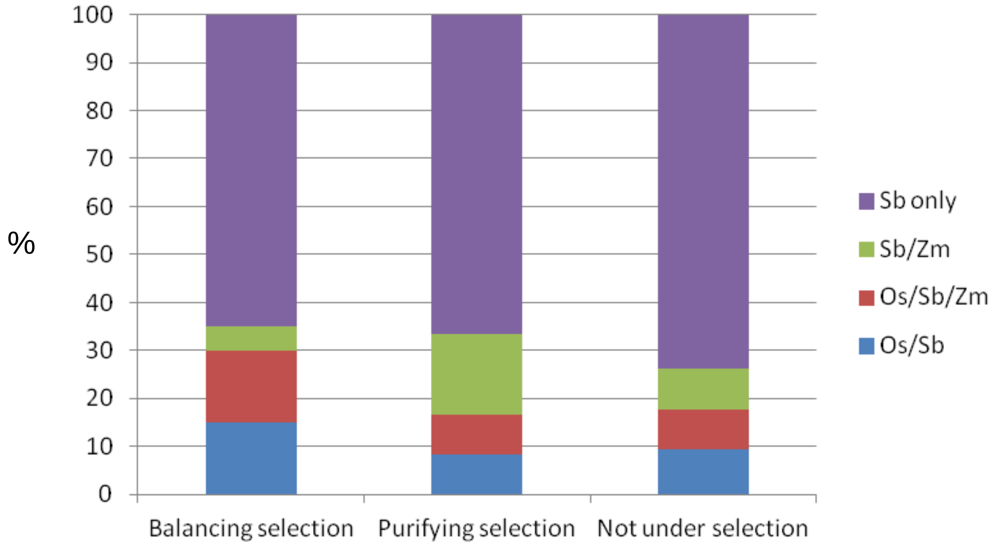

Figure S13. Proportion of cross species (Sb/Zm, Os/Sb/Zm and Os/Sb) NBS-encoding gene families in comparison to sorghum specific gene families for genes under purifying or balancing selection or under no selection.

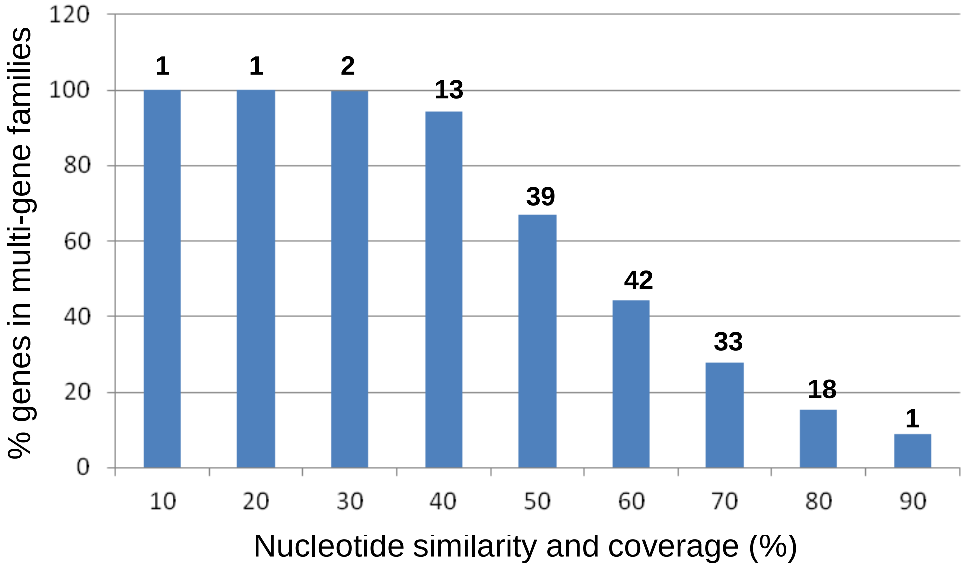

Figure S14. Percentage of genes in multi-gene families across different nucleotide similarity and gene coverage thresholds in sorghum. Numbers above each column indicate the total number of gene families identified at each threshold.

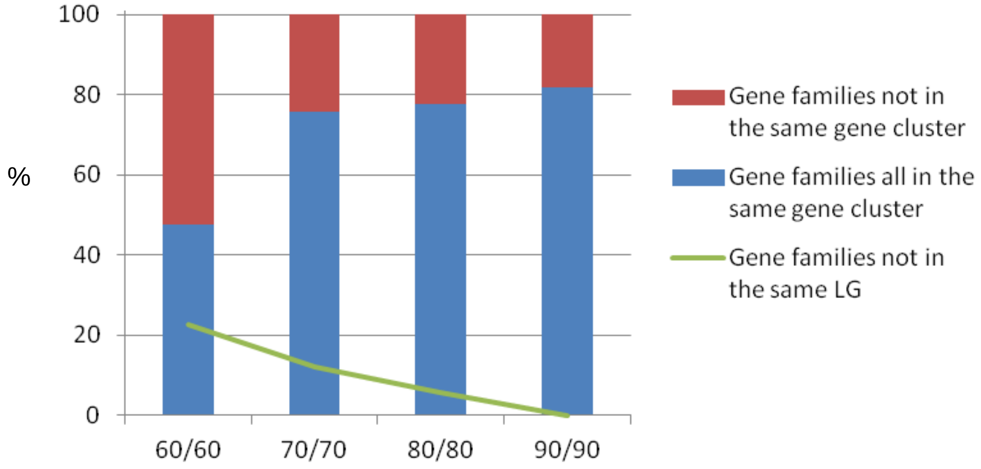

Figure S15. Comparison of gene family genome-wide distribution across thresholds in sorghum

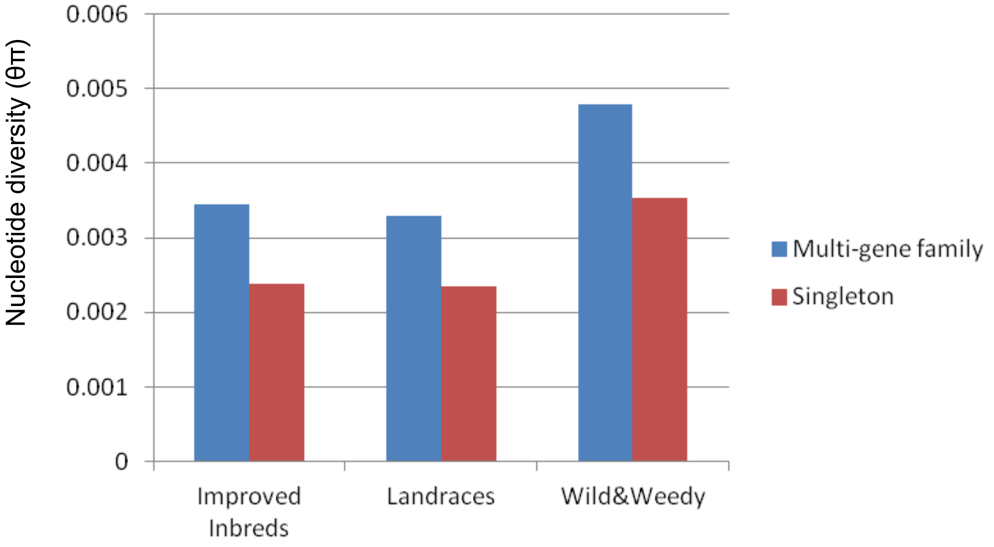

Figure S16. Diversity (θπ) of NBS-encoding genes across the three sorghum groups within paralogous multi-gene family versus singletons.

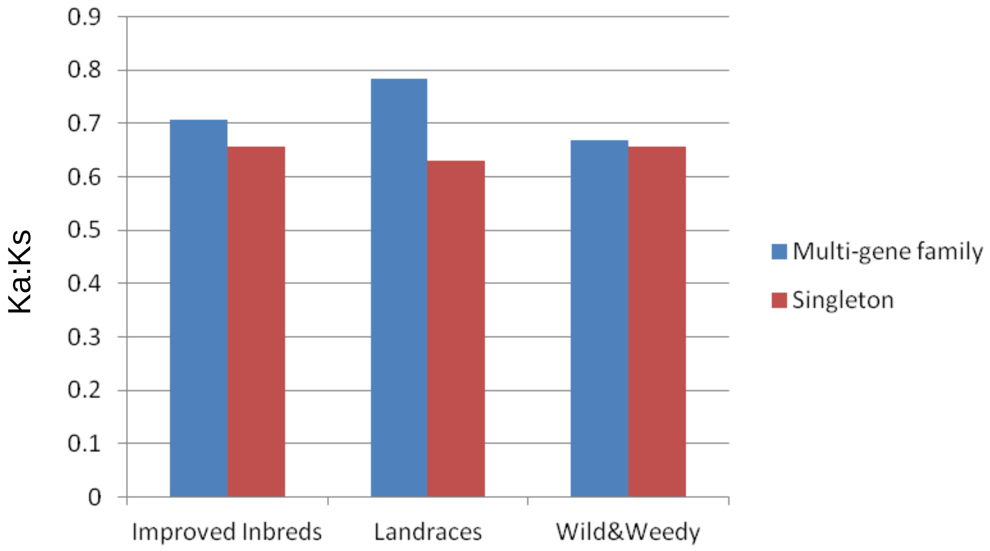

Figure S17. Comparison of Ka:Ks ratio in NBS singleton genes and multi-gene families across genotype groups in sorghum

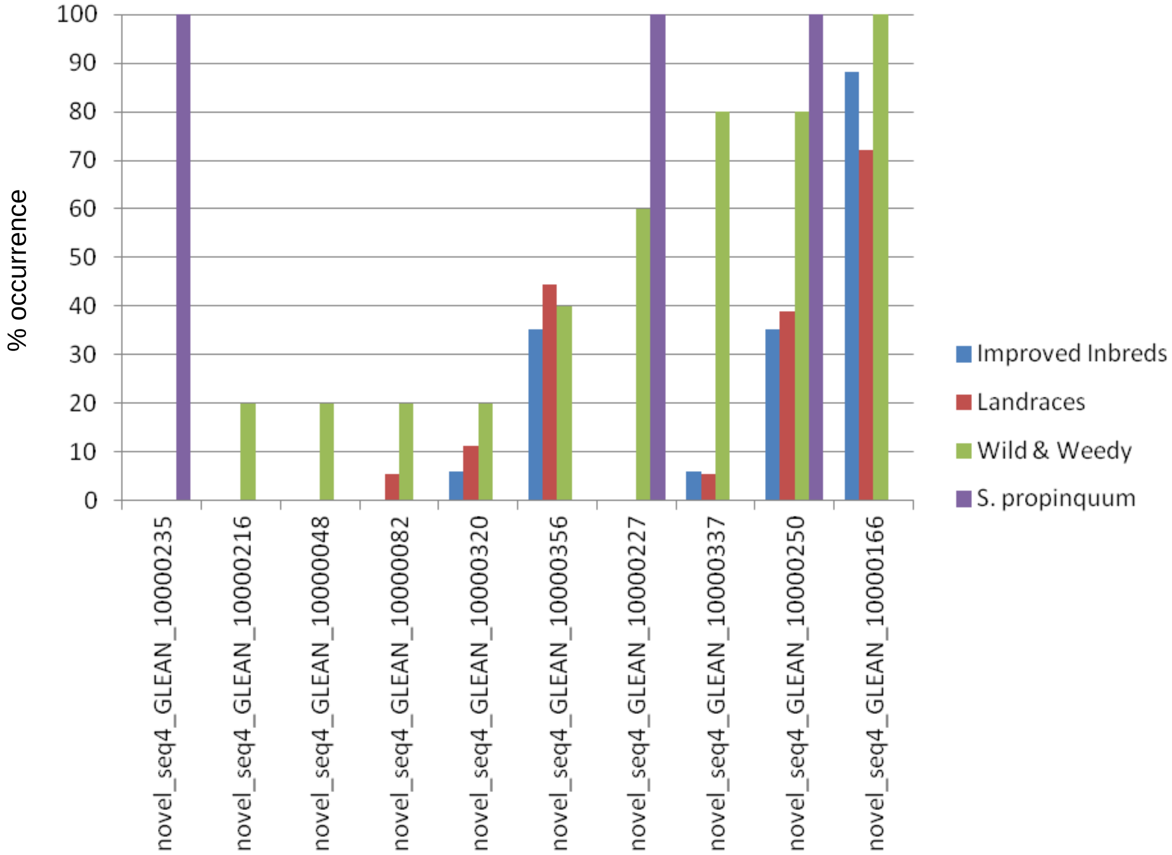

Figure S18. The occurrence of the ten NBS-encoding novel genes across the three *S. bicolor* groups, in addition to *S. propinquum*.

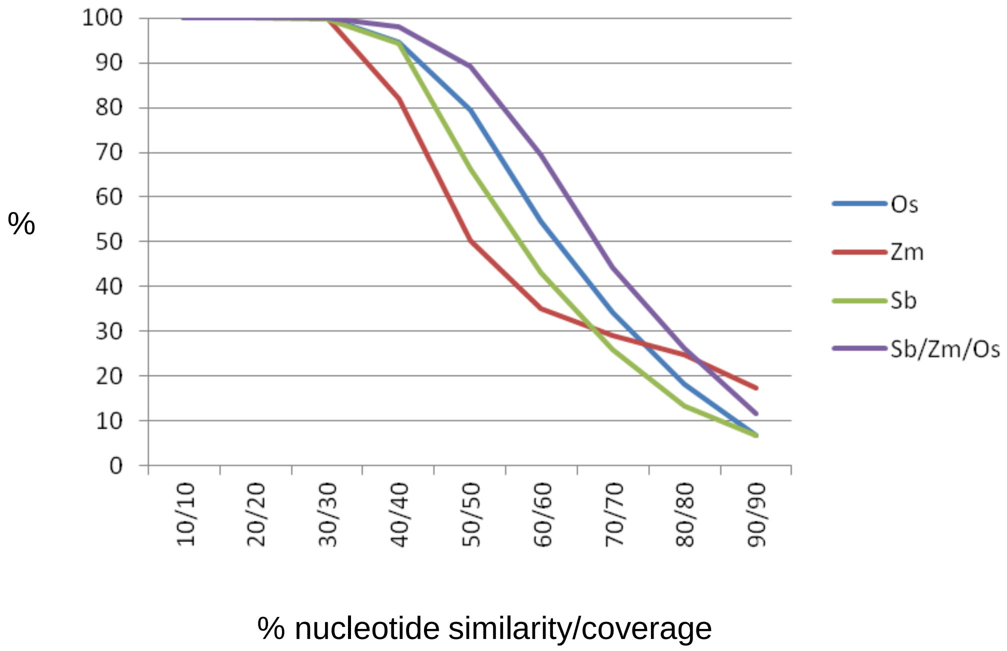

Figure S19. % of genes in multi-gene families across different nucleotide similarity/coverage thresholds and across species (Os: *Oryza sativa*; Zm: *Zea mays*; Sb: *Sorghum bicolor*)

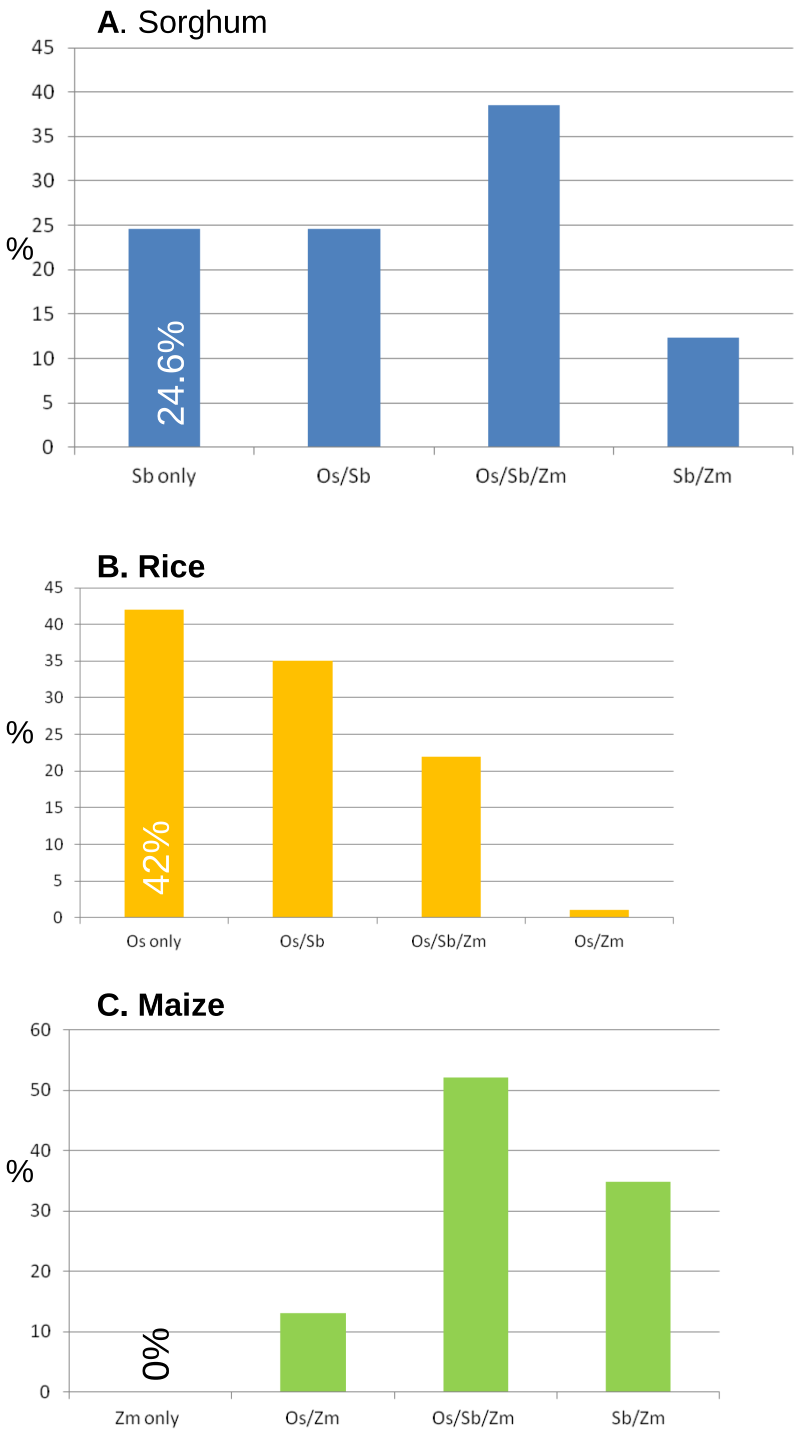

Figure S20. Proportion of NBS gene clusters syntenic across species

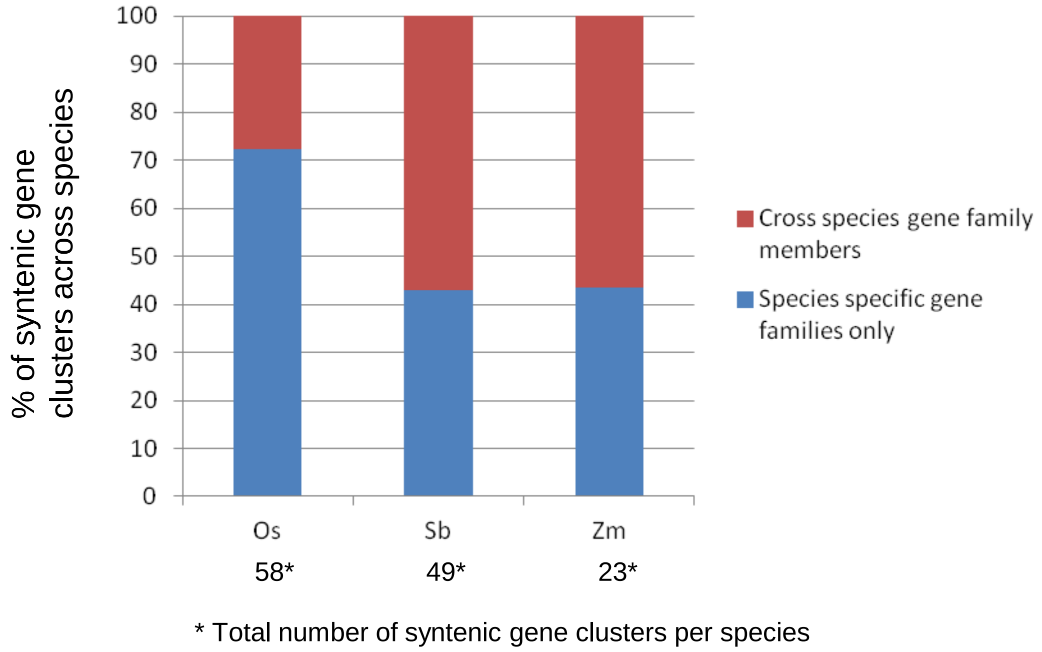

Figure S21. The proportion of syntenic gene clusters with species-specific gene families

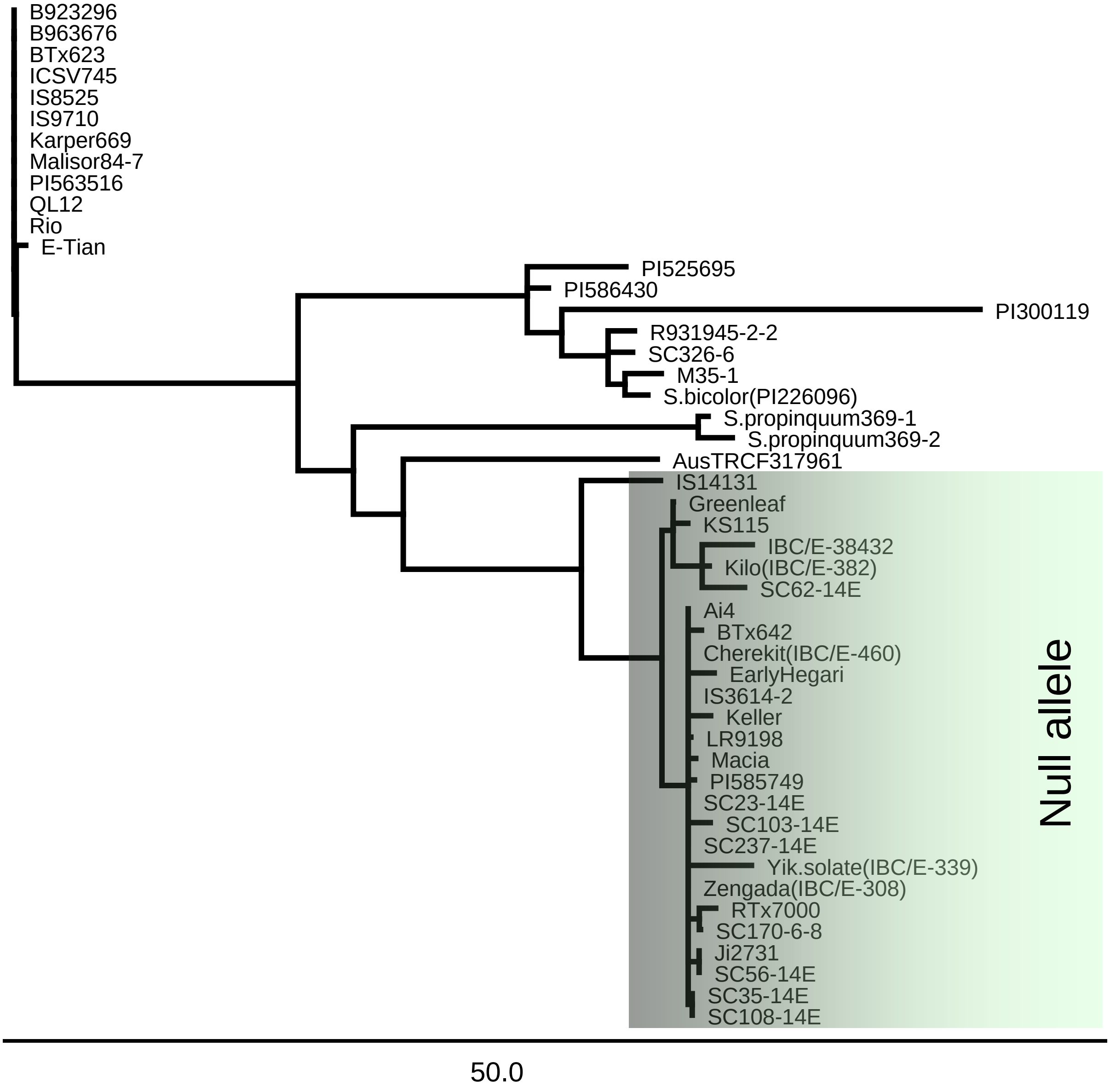

Figure S22. Gene tree for Sb08g005620 constructed using the neighbor-joining method with the Jukes-Cantor genetic distance model (Geneious version 6.1.4.) with null alleles highlighted

	# NBS genes in clusters	# singleton NBS genes	# clusters	Average # genes/cluster
SBI-01	6	12	3	2
SBI-02	45	10	13	3.46
SBI-03	5	11	2	2.5
SBI-04	0	8	0	0
SBI-05	98	11	19	5.16
SBI-06	10	6	3	3.33
SBI-07	18	11	5	3.6
SBI-08	35	10	12	2.92
SBI-09	9	8	3	3
SBI-10	12	6	5	2.4
Unassigned	0	15	0	0

Table S2. Details of the number of NBS-encoding genes per chromosome in sorghum; the number of NBS-encoding genes in gene clusters, the number of singleton NBS-encoding genes, the number of clusters and the average number of genes/cluster.

Input file	ML statistic (selection)	ML statistic (neutral)	DF	Likelihood ratio statistic	P value
1	388.514	340.273	17	96.482	3.97337E-13
2	365.501	340.235	17	50.532	3.49119E-05
3	362.123	340.157	17	43.932	0.0003505

Table S3A. Maximum-likelihood analysis of nucleotide polymorphism in a subset of 17 NBS-encoding candidate genes for domestication and improvement to determine whether a model of neutral or adaptive evolution best explained the patterns of nucleotide polymorphisms. The candidate genes were run 3 times with 38 neutral genes used for comparison in each run.

Input file	ML statistic (selection)	ML statistic (neutral)	DF	Likelihood ratio statistic	P value
1	494.632	452.323	23	84.600	5.62531E-09
2	496.839	452.303	23	89.072	1.017E-09
3	488.317	452.261	23	72.112	5.71499E-07

Table S3B. Maximum-likelihood analysis of nucleotide polymorphism in a subset of 23 NBS-encoding candidate genes for domestication and improvement to determine whether a model of neutral or adaptive evolution best explained the patterns of nucleotide polymorphisms. The candidate genes were run 3 times with 38 neutral genes used for comparison in each run.

	Improved Inbreds	Landraces	Wild & Weedy
% large effect SNPs	2.17	2.05	5.22
% frame-shifting indels	16.06	18.02	23.12

Table S4. The proportion of occurrence of large effect SNPs and frame-shifting indels in NBS-encoding genes in the three sorghum groups.

Novel gene ID	Hmm name	Hmm Acc	Bit score	E-value
novel_seq4_GLEAN_10000216	NB-ARC	PF00931.17	49.4	2.50E-13
novel_seq4_GLEAN_10000356	NB-ARC	PF00931.17	73.2	1.40E-20
novel_seq4_GLEAN_10000227	NB-ARC	PF00931.17	37.6	9.80E-10
novel_seq4_GLEAN_10000048	NB-ARC	PF00931.17	50.8	9.10E-14
novel_seq4_GLEAN_10000166	NB-ARC	PF00931.17	80.3	9.30E-23
novel_seq4_GLEAN_10000235	NB-ARC	PF00931.17	87.1	8.10E-25
novel_seq4_GLEAN_10000082	NB-ARC	PF00931.17	79	2.30E-22
novel_seq4_GLEAN_10000337	NB-ARC	PF00931.17	45.6	3.50E-12
novel_seq4_GLEAN_10000320	NB-ARC	PF00931.17	157.4	3.10E-46
novel_seq4_GLEAN_10000250	NB-ARC	PF00931.17	44.7	6.50E-12

Table S5. List of novel NBS-encoding genes identified previously [3], with details of the scores and e-values to PFAM00931.

Trait	Klein et al 2001 [4]	Mace & Jordan 2010 [5]	Mohan et al 2009 [6]	Mohan et al 2010 [7]	Nagy et al 2007 [8]	Parh et al 2008 [9]	Perumal et al 2009 [1]	Rami et al 1998 [10]	Tao et al 1998 [11]	Upadhyaya et al 2013 [12]	Upadhyaya et al 2013 [13]	**Grand Total**
Downy mildew resistance		1										**1**
Drechstera leaf blight resistance			1	1								**2**
Ergot resistance						6						**17**
Grain mold resistance	5							4			2	**11**
Milo disease resistance					1							**1**
Resistance to anthracnose	1			4			1			8		**14**
Rust resistance		1		2					4		5	**12**
Target leaf spot resistance			1	2								**3**
Zonate leaf spot resistance	1		1	3								**5**
**Grand Total**	**7**	**2**	**3**	**12**	**1**	**6**	**1**	**4**	**4**	**8**	**7**	**66**

Table S6. Summary of 9 fungal pathogen disease resistance traits identified across 12 studies

References

1. Perumal R, Menz MA, Mehta PJ, Katile S, Gutierrez-Rojas LA, Klein RR, Klein PE, Prom LK, Schlueter JA, Rooney WL, Magill CW: **Molecular mapping of Cg1, a gene for resistance to anthracnose (*Colletotrichum sublineolum*) in sorghum**. *Euphytica* 2009, **165**:597-606
2. Kellogg EA: **Floral displays: genetic control of grass inflorescences**. *Curr Opin Pl Biol* 2007, **10**: 26-31.
3. Mace ES, Tai S, Gilding EK, Li Y, Prentis PJ, Bian L, Campbell BC, Hu W, Innes DJ, Han X, Cruickshank A, Dai C, Frère C, Zhang H, Hunt CH, Wang X, Shatte T, Wang M, Su Z, Li J, Lin X, Godwin ID, Jordan DR, Wang J: **Whole genome sequencing reveals untapped genetic potential in Africa's indigenous cereal crop sorghum**. *Nat Comms* 2013, **4**: 2320.
4. Klein RR, Rodriguez-Herrera R, Schlueter JA, Klein PE, Yu ZH, Rooney WL: **Identification of genomic regions that affect grain-mould incidence and other traits of agronomic importance in sorghum.** *Theor Appl Genet* 2001, **102**:307-319.
5. Mace ES, Jordan DR: **Location of major effect genes in sorghum (*Sorghum bicolor* (L.) Moench).** *Theor Appl Genet* 2010, **121**: 1339-1356
6. Mohan SM, Madhusudhana R, Mathur K, Howarth CJ, Srinivas G, Satish K, Reddy RN, Seetharama N: **Co-localization of quantitative trait loci for foliar disease resistance in sorghum.** *Plant Breeding* 2009, **128**:532-535.
7. Mohan SM, Madhusudhana R, Mathur K, Chakravarthi DVN, Rathore S, Reddy RN, Satish K, Srinivas G, Mani NS, Seetharama N: **Identification of quantitative trait loci associated with resistance to foliar disaeases in sorghum [*Sorghum bicolor* (L.) Moench].** *Euphytica* 2010, **176**: 199-211
8. Nagy ED, Lee TC, Ramakrishna W, Xu ZJ, Klein PE, SanMiguel P, Cheng CP, Li JL, Devos KM, Schertz K, Dunkle L, Bennetzen JL: **Fine mapping of the Pc locus of *Sorghum bicolor*, a gene controlling the reaction to a fungal pathogen and its host-selective toxin.** *Theor Appl Genet* 2007, **114:** 961-970.
9. Parh DK, Jordan DR, Aitken EAB, Mace ES, Jun-ai , McIntyre CL, Godwin ID: **QTL analysis of ergot resistance in sorghum.** *Theor Appl Genet* 2008, **117**:369-382.
10. Rami JF, Dufour P, Trouche G, Fliedel G, Mestres C, Davrieux F, Blanchard P, Hamon P: **Quantitative trait loci for grain quality, productivity, morphological and agronomical traits in sorghum (*Sorghum bicolor* L. Moench).** *Theor Appl Genet* 1998, **97**:605-616
11. Tao YZ, Jordan DR, Henzell RG, McIntyre CL: **Identification of genomic regions for rust resistance in sorghum***.* *Euphytica* 1998, **103**:287-292
12. Upadhyaya HD, Wang Y-H, Sharma R, Sharma S: **Identification of genetic markers linked to anthracnose resistance in sorghum using association analysis**. *Theor Appl Genet* 2013, **126**: 1649-1657.
13. Upadhyaya HD, Wang Y-H, Sharma R, Sharma S: **SNP markers linked to leaf rust and grain mold resistance in sorghum**. *Mol Breeding* 2013, **32**: 451-462.
